# Western Diet Inhibits FUT7‐Mediated Treg Intestinal Homing to Disrupt the Homeostasis of Intestinal Epithelial Cells in Crohn's Disease

**DOI:** 10.1002/advs.202509541

**Published:** 2025-12-15

**Authors:** Qian Zhou, Xianfei Wang, Teng Ben, Chunping Zhu, Xinlong Lin, Yinmu Li, Xinyue Zhang, Yeling Chen, Yuchen Xuan, Huaiwen Chen, Ke Liu, Fang Wu, Yuexin Ren, Gao Tan

**Affiliations:** ^1^ Guangdong Provincial Key Laboratory of Gastroenterology Institute of Gastroenterology of Guangdong Province Department of Gastroenterology Nanfang Hospital Southern Medical University Guangzhou 510515 China; ^2^ Department of Gastroenterology Digestive Endoscopy Center Affiliated Hospital of North Sichuan Medical College Nanchong 637000 China; ^3^ Department of Gastroenterology Ganzhou Hospital Affiliated to Nanfang Hospital Southern Medical University Ganzhou 341099 China; ^4^ Sunlipo Biotech Research Center for Nanomedicine 3688 Tingwei Road Shanghai 201507 China; ^5^ Department of Gastroenterology The First Affiliated Hospital of Wenzhou Medical University Wenzhou 32500 China

**Keywords:** Crohn's disease, epithelial barrier, FUT7, treg cell, western diet

## Abstract

The Western diet (WD) is a potential risk factor for developing Crohn's disease (CD), characterized by disordered homeostasis of intestinal epithelial cells (IECs). However, how WD disrupts the IEC homeostasis remains unclear. Here, it is shown that WD disrupts the IEC homeostasis through inhibiting Fut7‐mediated Treg intestinal homing to weaken Treg and IEC signaling crosstalk. In this study, it is found that WD disrupted the IEC barrier, downregulated Fut7 expression in Tregs, and decreased colonic Tregs. The combined analyses of single‐cell RNA‐sequencing and spatial transcriptomics revealed that WD weakened the intestinal homing ability of Tregs and the signaling crosstalk between Tregs and IECs. Furthermore, Treg‐specific Fut7 knockout decreased colonic Tregs and aggravated the severity of colitis and IEC barrier disruption in the 2,4,6‐trinitrobenzenesulfonic acid‐induced mouse CD model, while upregulation of Fut7 in Tregs alleviated their severity. On top of this model, transfer of Tregs in vivo ameliorated colitis and IEC barrier disruption in the naive CD4^+^ T cell transfer model, but Fut7‐knockdown Tregs cannot do so. In addition, it is found that FUT7 expression in Tregs is downregulated in patients with active CD. Thus, these findings offer a novel insight into the pathogenesis of CD.

## Graphical abstract

In this study, we constructed a nanoparticle that was CD4 antibody‐mediated targeted, Fut7‐expressing plasmid‐loaded cationic liposome, namely CD4‐LDP‐Fut7. Upregulation of Fut7 expression in Tregs by using CD4‐LDP‐Fut7 can increase Treg homing to the intestine, thereby facilitating repair of the intestinal epithelial barrier and inhibiting inflammation in the intestine.

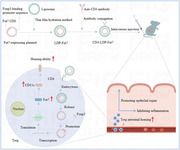

## 1 Introduction

Crohn's disease (CD), a refractory inflammatory disorder of the gastrointestinal tract, is a type of inflammatory bowel disease (IBD).^[^ ^1^ ^]^ CD is more prevalent in Western countries, such as the United States and Europe. The incidence of CD is four times higher in North America than in the Middle East or Asia, reflecting the fact that environmental factors, such as a high‐fat and high‐sugar Western diet (WD), drive the significant increase in CD incidence in developed countries.^[^ ^2^ ^]^ A recent multicenter study found a significant increase in the incidence of CD in countries with previously low prevalence, and this increase paralleled WD adoption in these countries, suggesting that WD is intensively associated with increased CD risk.^[^ ^3^ ^]^ However, the underlying mechanism between WD and CD development is still largely unclear.

Accumulating evidence indicates that CD pathogenesis is involved in the impairment of the intestinal epithelial cell (IEC) barrier, a vital physical barrier against gut microbial activation of the mucosal immune system.^[^ ^4^, ^5^, ^6^ ^]^ Current evidence suggests that an exaggerated mucosal immune response to the gut microbes in CD is due to disruption of the IEC barrier.^[^ ^4^ ^]^ The tight junction, mainly composed of Occludin and ZO‐1, is a critical structure of the IEC barrier,^[^ ^7^, ^8^, ^9^ ^]^ and its dysfunction can cause gut leakage and inflammation, which is a vital factor in the development of gastrointestinal diseases such as CD.^[^ ^6^, ^10^ ^]^ However, the etiology of impaired IEC tight junction and barrier, and its triggering CD mechanisms, remains enigmatic. As we know, impaired IECs can be repaired by immune cells, and the normal signaling crosstalk between immune cells and IECs is crucial for maintaining the IEC homeostasis.^[^ ^11^, ^12^ ^]^ These immune cells mainly refer to Tregs that play a critical role in maintaining tissue homeostasis through controlling excessive immune responses, restraining inflammation, and promoting tissue regeneration and repair.^[^ ^13^ ^]^ However, whether and how WD regulates the signaling crosstalk between Tregs and IECs to affect the IEC homeostasis in CD remains to be determined.

The ability of T lymphocytes to home to peripheral tissues is critically dependent on Sialylated Lewis X (CD15s) on the T‐cell surface, a terminal oligosaccharide linked to transmembrane glycoproteins, such as CD44 and CD43.^[^ ^14^ ^]^ CD15s is the core recognition epitope of L‐, P‐, and E‐selectin, and their binding plays a critical role in mediating lymphocyte homing.^[^ ^15^, ^16^ ^]^ CD15s synthesis is specifically catalyzed by α(1,3)Fucosyltransferase VII (FucT VII, FUT7).^[^ ^15^, ^16^ ^]^ Previous studies revealed that Fut7 imparts L‐, P‐, and E‐selectin binding activity to ligands and controls the ability of T lymphocytes to home to inflammatory lesions and peripheral lymph nodes in mice.^[^ ^16^, ^17^ ^]^ A previous report showed that the number of Tregs is relatively insufficient in the inflamed intestinal mucosa of patients with CD.^[^ ^18^ ^]^ However, it is unknown whether this insufficiency is attributed to FUT7 downregulation in Tregs.

In this study, we hypothesized that WD disrupts the IEC barrier through downregulating FUT7 expression to decrease Treg homing to the gut. To test this hypothesis, we first constructed a WD model as described in our previous description^[^ ^19^ ^]^ and found that WD not only disrupted the IEC barrier but also decreased colonic Tregs and their Fut7 expression. Subsequently, the combined analysis of scRNA‐seq and ST revealed that WD inhibited the intestinal homing ability of Tregs and weakened the signaling crosstalk between Tregs and IECs. Next, the transfer model of colitis and the TNBS model were used to further strengthen our finding that the inhibition of colitis and the impairment of the IEC barrier by Tregs require Fut7 expression. Moreover, we constructed a nanoparticle that specially targets Tregs to express Fut7 and found that this nanoparticle significantly alleviated colitis and promoted IEC repair in the WD and TNBS models. Importantly, we explored the clinical effects of FUT7 in Tregs and found that the expression of FUT7 in Tregs was decreased in patients with active CD. Therefore, these findings might offer an insight into CD pathogenesis.

## 2 Results

### 2.1 WD Decreases Fut7 Expression in Tregs, Treg Homing to the Intestine, and Disrupts the IEC Barrier in Mice

**Figure 1:**
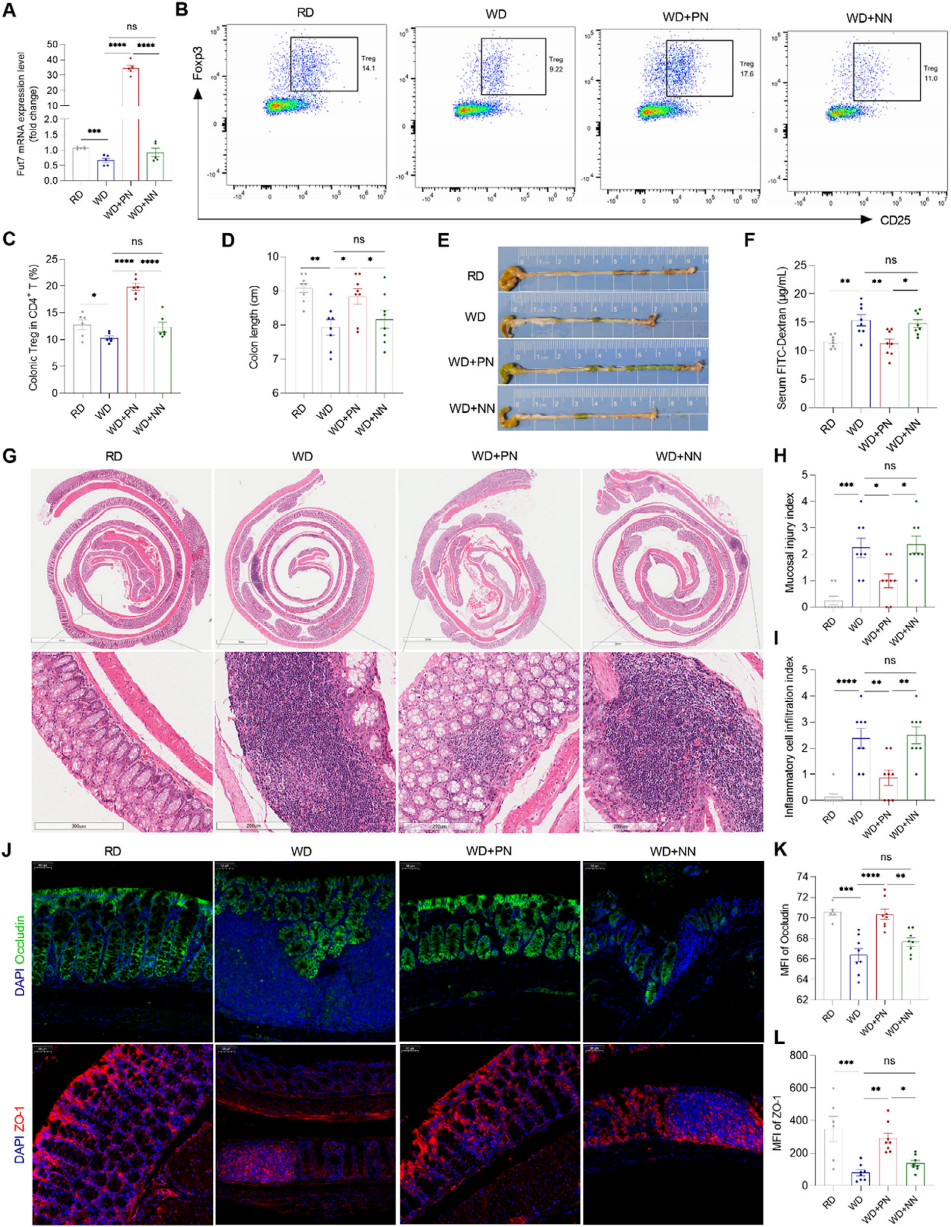
Upregulation of Fut7 in Tregs improves WD‐induced intestinal barrier disruption in mice. *A*–*L*) Littermate WT mice were divided into four groups (*n* = 8/group). They were fed with a normal diet (RD) in group 1 and a western diet (WD) in groups 2–4 for 17 weeks. In week 17, the positive nanoparticle (PN) CD4‐LDP‐Fut7 and the negative nanoparticle (NN) CD4‐LDP‐NC were given by tail intravenous injection at a dose of 240 ng/g once every two days for a total of three times in groups 3 and 4, respectively. (*A*) Fut7 mRNA expression in FACS‐sorted Tregs from the spleen. (*B*) Representative flow cytometric plots of colonic CD25^+^ Foxp3^+^ Tregs. (*C*) The frequency of CD25^+^ Foxp3^+^ Tregs in CD4^+^ T cells. (*D*) Colon length. (*E*) Representative gross morphology of the colon. (*F*) After 4 h of fasting, the mice were given FITC‐conjugated dextran by gavage administration for 4 h, and subsequently, the concentration of FITC‐dextran in blood serum was measured. (*G*) Representative intestinal H&E staining. (*H*) Mucosal injury index. (*I*) Inflammatory cell infiltration index. (*J*) Representative immunofluorescence images of Occludin and ZO‐1 immunostaining in colonic tissues. (*K, L*) MFI of Occludin and ZO‐1 was quantified by ImageJ software. The data shown are representative of three independent experiments. (*A, C, D, F, K, L*) one‐way ANOVA or (*H, I*) Kruskal‐Wallis test; ^*^ *P* < 0.05, ^**^ *P* < 0.01, ^***^ *P* < 0.001, ^****^ *P* < 0.0001; ns, not significant. FACS, fluorescence‐activated cell sorting; MFI, mean fluorescence intensity; WT, wild‐type.

To test the effect of WD on regulating Fut7 expression in Tregs, controlling Treg homing to the intestine, and disrupting the IEC barrier, we first fed wild‐type mice a regular diet (RD) or WD and found that compared with RD feeding, WD feeding downregulated the mRNA expression of Fut7 in Tregs and decreased the frequency of colonic Tregs among CD4^+^ T cells (**Figure** **1**A–C). Single‐cell RNA sequencing revealed lower Fut7 expression in Tregs from the WD group than in those from the regular diet (RD) group (Figure S1, Supporting Information). In addition, compared with RD feeding, WD feeding disrupted the IEC barrier and triggered colitis, as reflected by increased colon shortening, IEC damage, and histological changes, as well as increased serum FITC‐Dextran and reduced levels of colonic tight junction proteins Occludin and ZO‐1 in WD‐fed mice (Figure 1D–L).

To explore whether WD affects the ability of Tregs to home to the intestine, we next performed TCR‐seq on colorectal tissues and peripheral blood from RD‐ and WD‐fed mice and compared the similarity of TCR in Tregs between the colorectal tissues and peripheral blood from the same mouse by Morphisita and overlap analysis (Figure S2A,B, Supporting Information). Compared with RD feeding, WD feeding decreased TCR similarity in Tregs between the colorectal tissues and peripheral blood, as reflected by reduced morisita and overlap index in WD‐fed mice. This result suggests that WD can impair the ability of Tregs to home to the intestine.

**Figure 2:**
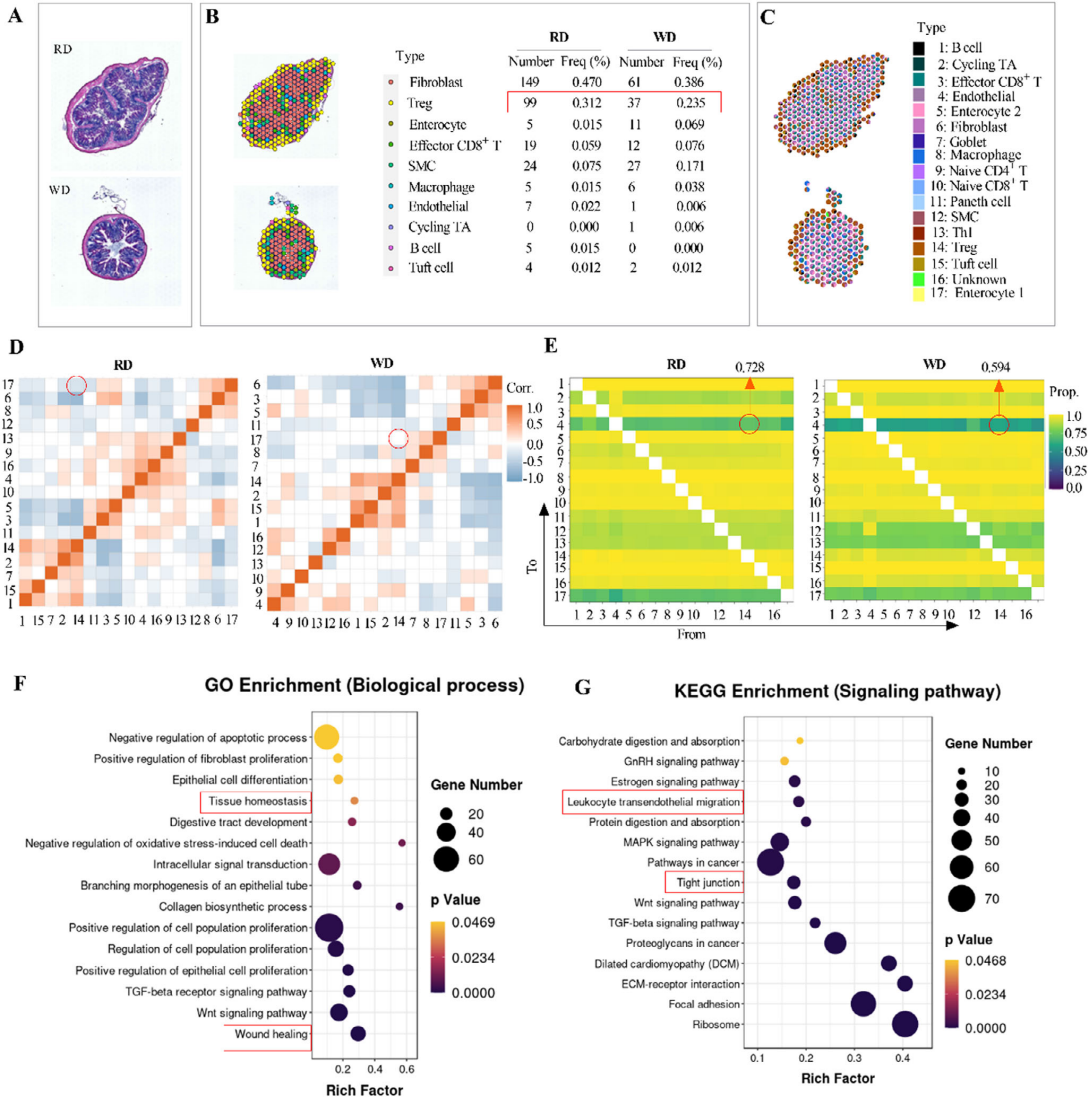
Integration of scRNA‐seq and ST analyses of the colon from RD‐ and WD‐fed mice. *A–G*) Colorectum tissues were collected from littermate WT mice fed with a regular normal diet (RD) and Western diet (WD) for 16 weeks, then spatial transcriptomics (ST) and single‐cell RNA‐sequencing (scRNA‐seq) analyses were integrated. (*A*) H&E staining of transcriptome sections. (*B*) Spot clustering of transcriptome sections. (*C‐G*) Integration of ST regions and cell subsets of scRNA‐seq data using the MIA method. (*D*) Correlation analysis of the cell‐cell interaction. (*E*) Colocalization analysis of major cell types in the intestine. (*F, G*) GO and KEGG enrichment analysis of differentially expressed genes (DEGs) in the Treg cluster between the two groups. Dot size represents the gene number of DEGs, and the corresponding color represents its *P* value. MIA, multimodal intersection analysis; GO, gene ontology; KEGG, Kyoto encyclopedia of genes and genomes; WT, wild‐type.

To investigate whether WD disrupts the IEC barrier through impairing the cell‐cell interaction between Tregs and IECs, we subsequently performed spatial transcriptomics (ST) analysis on colorectal tissues from RD‐ and WD‐fed mice, and their ST sections were stained with H&E (**Figure** **2**A). According to the ST sequencing data, spots in the spatial sections were divided into 10 regions (Figure 2B). Compared with RD feeding, WD feeding decreased the number and frequency of colonic Tregs (Figure 2B). We then used MIA, a hypergeometric distribution test method,^[^ ^20^ ^]^ to integrate the results of ST and scRNA‐seq. The association between the single‐cell clusters and spatial regions was analyzed to identify cell types distributed in each spot (Figure 2C). Correlation analysis revealed that WD weakened cell‐cell interaction from Tregs to the Enterocyte 1 cluster, a major class of IECs (Figure 2D). Cellchat analysis showed that a WD substantially attenuates signaling from Tregs to IECs, and the disruption of Treg–IEC crosstalk occurs independently of other CD4⁺T cell populations (Figure S3, Supporting Information). In addition, WD reduced the colocalization of Tregs and endothelial cells (Figure 2E). Moreover, enriched biological process terms in Tregs included tissue homeostasis and wound healing (Figure 2F), while KEGG analysis in Tregs revealed the functional enrichment of leukocyte trans‐endothelial migration and tight junction (Figure 2G). Together, these results suggest that WD disrupts the IEC barrier by decreasing Treg homing to the intestine.

### 2.2 Upregulation of Fut7 in Tregs Ameliorates WD‐Induced Colonic Treg Decreases and Disruption of the IEC Barrier in Mice

To determine whether WD decreases Treg intestinal homing through downregulating Fut7 expression in Tregs, thereby disrupting the IEC barrier, we first constructed the nanoparticle CD4‐Lipid‐Polymeric Drug‐delivery system (LDP)‐Fut7 that consists of CD4 antibody‐mediated targeted, fut7‐expressing plasmid‐loaded cationic liposomes and specially targets Tregs to express Fut7, as shown in Figure S4 (Supporting Information). In the Fut7‐expressing plasmid, the promoter of the plasmid itself was cut off and replaced with the mouse Foxp3‐binding promoter sequence, followed by the coding sequences (CDS) of the mouse Fut7 gene and the enhanced green fluorescent protein (EGFP) gene, which were inserted into the plasmid in succession. In this study, we used the nanoparticle CD4‐LDP‐NC as a negative control, whose only difference from CD4‐LDP‐Fut7 was that the Fut7 CDS was not inserted. We found that CD4‐LDP‐Fut7 and CD4‐LDP‐NC can be activated in Tregs, but not in non‐Tregs (Figure S5, Supporting Information). In addition, the size, zeta potential, and plasmid encapsulation efficacy of the two nanoparticles were essentially consistent (Table S4, Supporting Information).

The nanoparticles did not cause discernible tissue damage in any of the main organs (Figure S6, Supporting Information). Fluorescence imaging in mice showed that the nanoparticles mainly accumulated in the small intestine (SI), colon, spleen, pancreas, kidneys, liver, and lung (Figure S7, Supporting Information). We then administered both nanoparticles to WD‐fed mice and observed that, compared with CD4‐LDP‐NC, CD4‐LDP‐Fut7 significantly increased Fut7 mRNA expression in Tregs and elevated the frequency of colonic Tregs among CD4⁺ T cells (Figure 1A–C). Furthermore, CD4‐LDP‐Fut7 substantially alleviated WD‐induced colitis and damage to the IEC barrier relative to CD4‐LDP‐NC, as indicated by reduced colon shortening, less IEC injury, and improved histology, along with lower serum FITC‐Dextran levels and higher colonic expression of the tight junction proteins Occludin and ZO‐1 (Figure 1D–L). These findings suggest that upregulating Fut7 in Tregs helps restore the WD‐induced disruption of the intestinal epithelial barrier.

### 2.3 Fut7 Confers the Ability of Tregs to Alleviate Disruption of the IEC Barrier in the T Cell Transfer Model of Colitis

**Figure 3:**
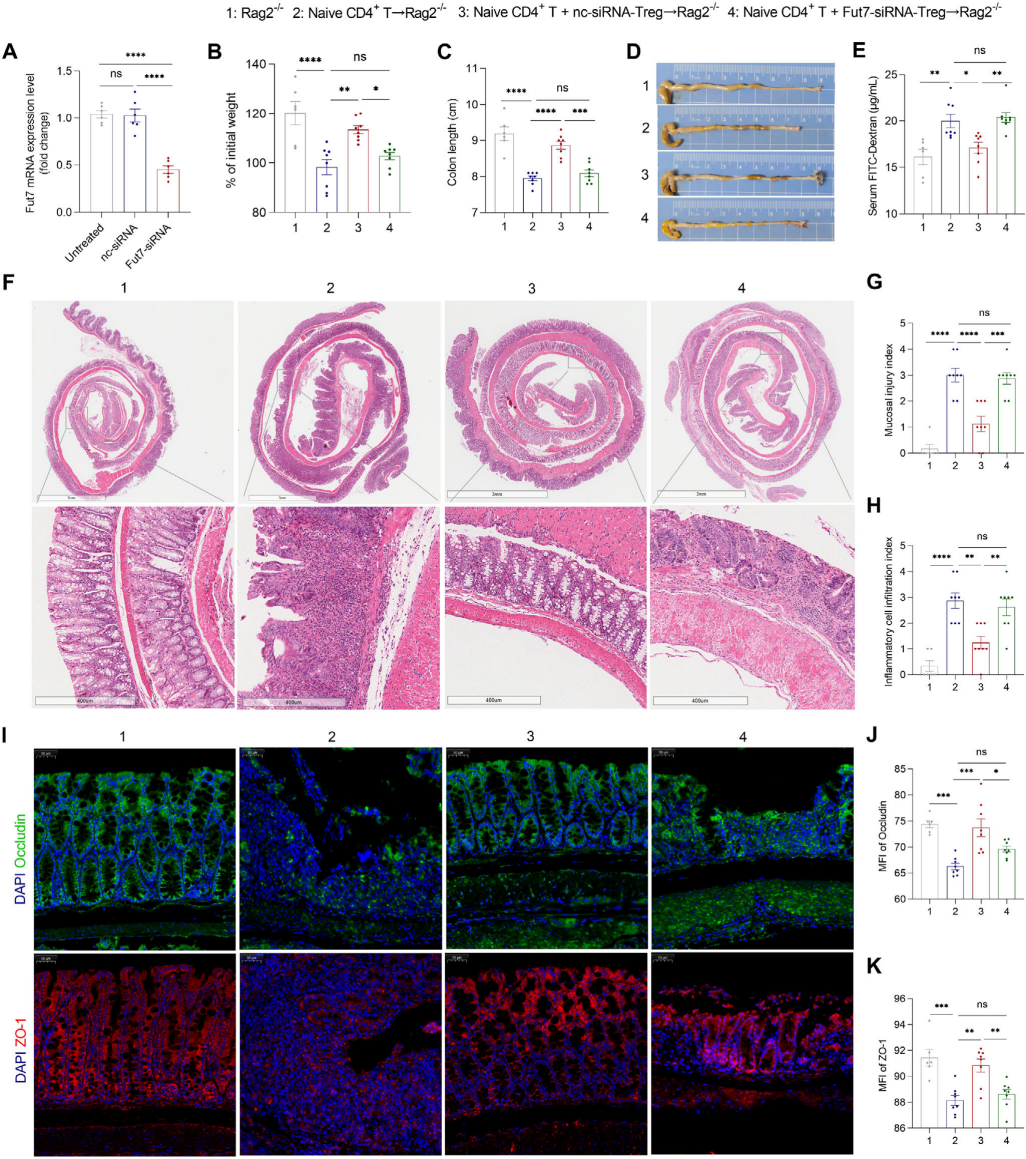
Fut7 confers Tregs to ameliorate the intestinal barrier in the adoptive transfer model. *A*) FACS‐sorted splenic CD4^+^ CD25^+^ CD127^low^ Tregs from WT mice were transfected with *Fut7*‐siRNA and negative control (nc)‐siRNA for 48 h in vitro, and subsequently, transfection efficiency was tested by real‐time PCR. *B*–*K*) *Rag*2^−/−^ mice were divided into four groups (n = 6–8/group). In group 1, the mice were not treated. In groups 2–4, the mice were given FACS‐sorted naive CD4^+^ CD45RB^high^ CD25^low^ T cells by tail intravenous injection for 6 weeks to induce a T cell transfer colitis model. In groups 3 and 4, FACS‐sorted splenic CD4^+^ CD25^+^ CD127^low^ Tregs were transfected with *Fut7*‐siRNA and nc‐siRNA for 48 h in vitro, and these Tregs, together with naive T cells, were injected into *Rag*2^−/−^ mice. (*B*) Mouse weight changes. (*C*) Representative gross morphology of the colon. (*D*) Colon length. (*E*) After 4 h of fasting, the mice were given FITC‐conjugated dextran by gavage administration for 4 h, and subsequently, the concentration of FITC‐dextran in blood serum was measured. (*F*) Representative intestinal H&E staining. (*G*) Mucosal injury index. (*H*) Inflammatory cell infiltration index. (*I*) Representative immunofluorescence images of Occludin and ZO‐1 immunostaining in colonic tissues. (*J, K*) MFI of Occludin and ZO‐1 was quantified by ImageJ software. The data shown are representative of three independent experiments. (*A, B, C, E, J, K*) one‐way ANOVA or (*G, H*) Kruskal‐Wallis test; ^*^ *P* < 0.05, ^**^ *P* < 0.01, ^***^ *P* < 0.001, ^****^ *P* < 0.0001; ns, not significant. FACS, fluorescence‐activated cell sorting; MFI, mean fluorescence intensity; WT, wild‐type.

To further verify the role of Fut7 in Tregs in repairing disruption of the IEC barrier in colitis, we used the naive CD4+ T cell transfer model of colitis.^[^ ^21^ ^]^ Tregs were isolated from wild‐type mice by flow cytometry, then transfected with Fut7‐siRNA to knock down Fut7 expression in vitro (**Figure** **3**A), and subsequently transferred into mice in this model. Compared with control mice, *Rag2* ^−/−^ mice receiving naive CD4+ T cells developed colitis, resulting in disruption of the IEC barrier, as reflected by increased colon shortening, IEC damage, and histological changes, as well as increased serum FITC‐Dextran and reduced levels of tight junction proteins Occludin and ZO‐1 in the colons (Figure 3B–K). In this colitis model, Tregs transfected with Fut7‐siRNA did not inhibit colitis or repair the IEC barrier, while Tregs transfected with negative control (nc)‐siRNA that did not knock down Fut7 expression could (Figure 3A–K). These results suggest that the repair of disruption of the IEC barrier by Tregs in this colitis model requires sufficient Fut7 expression.

### 2.4 Fut7 Deletion in Tregs Increases TNBS‐Induced Colitis and Disruption of the IEC Barrier in Mice

**Figure 4:**
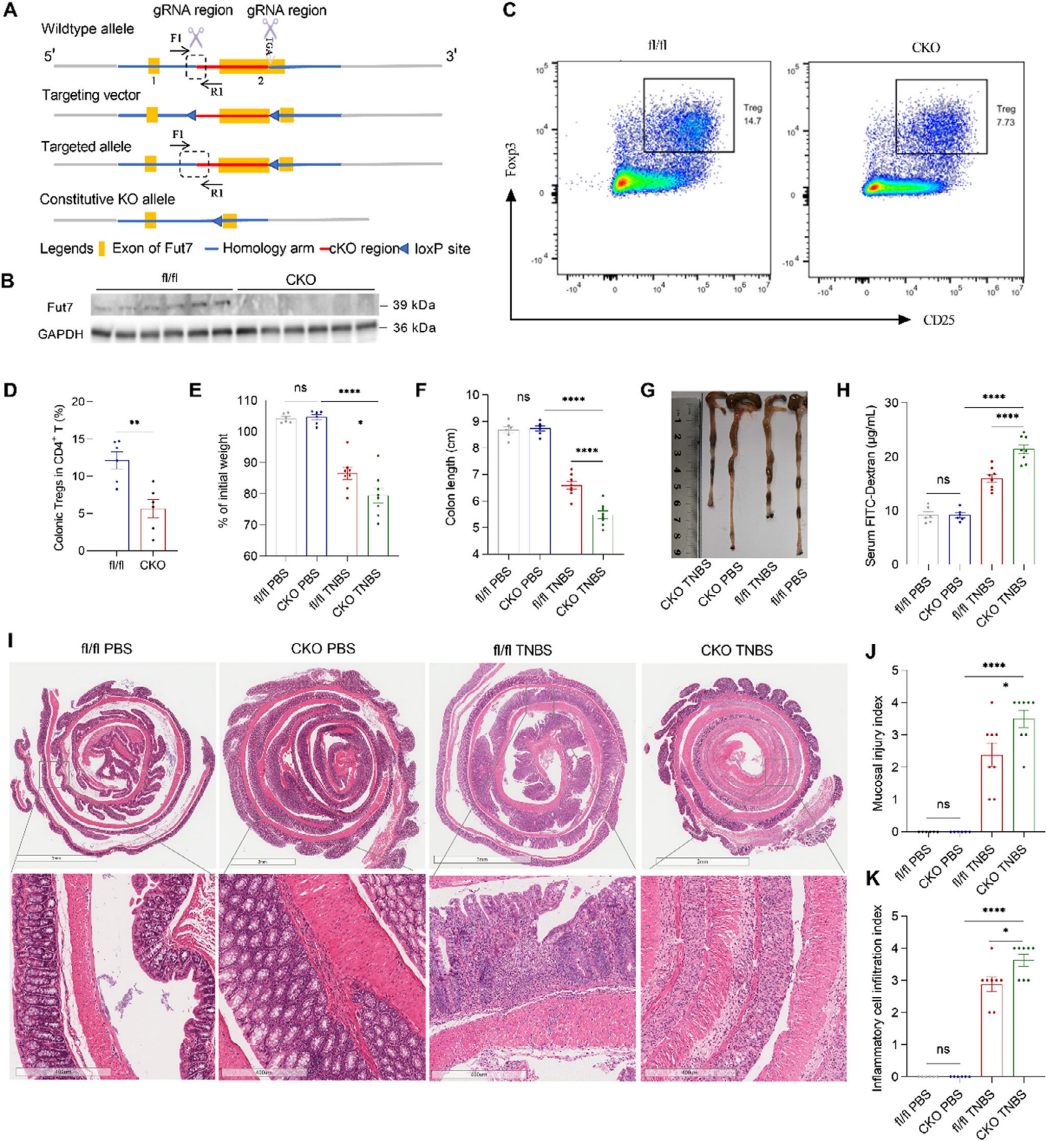
Specific deletion of Fut7 in Tregs deteriorates TNBS‐induced intestinal barrier disruption in mice. *A*) *fut7* ^fl/fl^ *foxp3* ^cre^ mice with conditional knockout (CKO) of the *fut7* gene in Tregs were generated as shown in the schematic diagram. *B*) The knockout efficiency was determined by Western blot analysis of Fut7 expression in FACS‐sorted splenic Tregs. *C*) Representative flow cytometric plots of colonic CD25^+^ Foxp3^+^ Tregs. *D*) The frequency of CD25^+^ Foxp3^+^ Tregs in CD4^+^ T cells. *E–K*) CKO and *fut7* ^fl/fl^ littermates were intrarectally injected with TNBS to induce colitis (n = 6–8/group). (*E*) Mouse weight changes. (*F*) Colon length. (*G*) Representative gross morphology of the colon. (*H*) After 4 h of fasting, the mice were given FITC‐conjugated dextran by gavage administration for 4 h, and subsequently, the concentration of FITC‐dextran in blood serum was measured. (*I*) Representative intestinal H&E staining. (*J*) Mucosal injury index. (*K*) Inflammatory cell infiltration index. Data shown in C–K are representative of three independent experiments. (*D*) unpaired Student's *t* test, (*E*, *F, H*) one‐way ANOVA, or (*J, K*) Kruskal‐Wallis test; ^*^ *P* < 0.05, ^**^ *P* < 0.01, ^****^ *P* < 0.0001; ns, not significant. FACS, fluorescence‐activated cell sorting; TNBS, 2,4,6‐trinitrobenzenesulfonic acid.

To confirm the role of Fut7 in controlling Treg homing to the colon, we first generated *fut7^fl/fl^ Foxp3^cre^* mice with Treg‐conditional knockout (CKO) of *fut7* and *fut7^fl/fl^* littermate controls (fl/fl) (**Figure** **4**A,B) and found that the frequency of colonic Tregs among CD4^+^ T cells was significantly lower in the CKO mice than in the fl/fl controls (Figure 4C,D), suggesting that Fut7 controls the ability of Treg homing to the colon. To further explore the effect of Fut7 in Tregs on the IEC barrier in the pathogenesis of CD, CKO mice and fl/fl controls were challenged with 2,4,6‐trinitrobenzenesulfonic acid (TNBS) to induce a CD‐like model, since the TNBS‐induced model simulates the clinical pathology of human CD.^[^ ^22^ ^]^ We found that the CKO mice were more sensitive to TNBS‐induced colitis and disruption of the IEC barrier than the controls were, as reflected by increased weight loss, colon shortening, and serum FITC‐Dextran (Figure 4E–H). Furthermore, histological examination revealed increased TNBS‐induced colonic mucosal inflammation, crypt loss, and epithelium damage in the CKO mice compared with the controls (Figure 4I–K).

### 2.5 Upregulation of Fut7 Expression in Tregs Improves TNBS‐Induced Colitis and Disruption of the IEC Barrier in Mice

**Figure 5:**
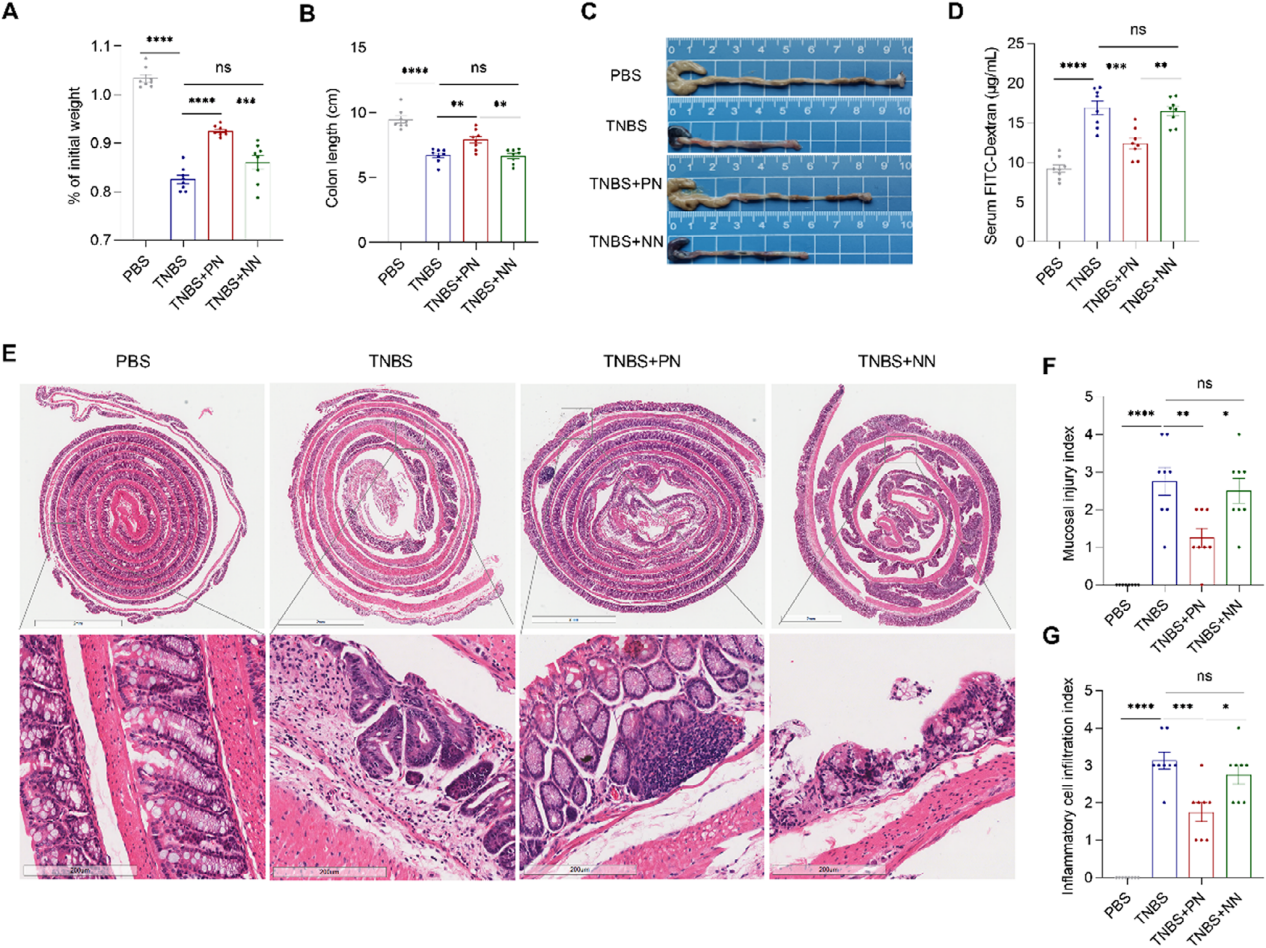
Upregulation of Fut7 in Tregs alleviates TNBS‐induced intestinal barrier disruption in mice. *A–G*) Littermate WT mice were intrarectally injected with TNBS to induce colitis. On days 1, 2, and 3, the positive nanoparticle (PN) CD4‐LDP‐Fut7 and the negative nanoparticle (NN) CD4‐LDP‐NC were given by tail intravenous injection at a dose of 240 ng/g for three times (n = 8/group). (*A*) Mouse weight changes. (*B*) Colon length. (*C*) Representative gross morphology of the colon. (*D*) After 4 h of fasting, the mice were given FITC‐conjugated dextran by gavage administration for 4 h, and subsequently, the concentration of FITC‐dextran in blood serum was measured. (*E*) Representative intestinal H&E staining. (*F*) Mucosal injury index. (*G*) Inflammatory cell infiltration index. The data shown are representative of three independent experiments. (*A*, *B, D*) one‐way ANOVA or (*F, G*) Kruskal‐Wallis test; ^*^ *P* < 0.05, ^**^ *P* < 0.01, ^***^ *P* < 0.001, ^****^ *P* < 0.0001; ns, not significant. TNBS, 2,4,6‐trinitrobenzenesulfonic acid; WT, wild‐type.

To explore whether upregulation of Fut7 expression in Tregs will be a useful therapeutic approach for patients with CD, we used the two nanoparticles CD4‐LDP‐Fut7 and CD4‐LDP‐NC in the TNBS‐induced colitis model. Compared with CD4‐LDP‐NC, CD4‐LDP‐Fut7 protected mice from TNBS‐induced colitis and disruption of the IEC barrier, as reflected by decreased weight loss, colon shortening, and serum FITC‐Dextran (**Figure** **5**A–D). Furthermore, histological examination revealed decreased TNBS‐induced colonic mucosal inflammation, crypt loss, and epithelium damage in the CD4‐LDP‐Fut7 group compared with the CD4‐LDP‐NC group (Figure 5E–G). These results indicate that CD4‐LDP‐Fut7 can significantly inhibit intestinal inflammatory responses and repair the IEC barrier in the mouse CD model.

### 2.6 FUT7 Expression in Tregs and CD15s^+^ Tregs is Decreased in Active CD Patients

**Figure 6:**
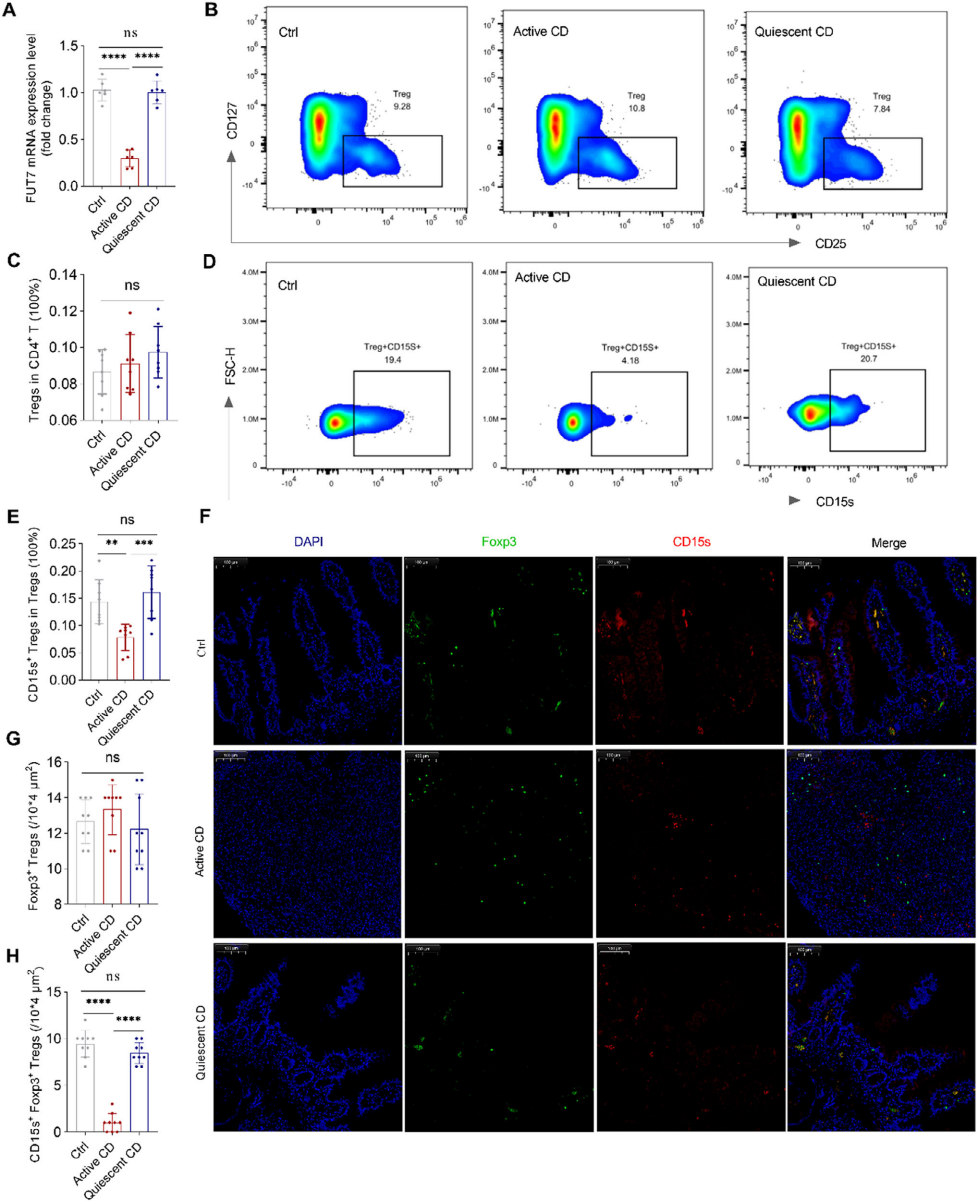
FUT7 expression in Tregs and CD15s^+^ Tregs is decreased in patients with active CD. *A*) Fut7 mRNA expression in FACS‐sorted Tregs from the blood of patients with active CD (n = 6), quiescent CD (n = 6), and healthy controls (n = 6). *B–E*) PBMCs were isolated from the blood of patients with active CD (n = 8), quiescent CD (n = 8), and healthy controls (n = 8) by density centrifugation, and then CD4^+^ CD25^+^ CD127^low^ Tregs and CD15s^+^ Tregs were analyzed by flow cytometry. (*B, D*) Representative flow cytometric plots. (*C*) The frequency of CD25^+^ CD127^low^ Tregs in CD4^+^ T cells. (*E*) The frequency of CD15s^+^ Tregs in Treg cells. (*F* to *H*) Fresh colonic mucosa from active CD (*n* = 9), quiescent CD (*n* = 9), and healthy controls (*n* = 9) were fixed in 4% paraformaldehyde and subjected to immunofluorescence staining using anti‐human antibodies targeting Foxp3 and CD15s. (*F*) Representative immunofluorescence images. (*G, H*) The number of indicated Treg subsets. (*A, C, E, G, H*) One‐way ANOVA; ^**^ *P* < 0.01, ^***^ *P* < 0.001, ^****^ *P* < 0.0001; ns, not significant. FACS, fluorescence‐activated cell sorting; PBMC, peripheral blood mononuclear cell.

To determine the clinical significance of FUT7 in Tregs in patients with CD, we first assessed the expression levels of FUT7 in FACS‐sorted Tregs from the blood of patients with active CD, quiescent CD, and healthy controls. The mRNA levels of FUT7 in Tregs were significantly lower in active CD patients, but not in quiescent CD patients, than in healthy controls (**Figure** **6**A). Consistently, we found that FUT7 expression levels in Tregs were negatively correlated with CDAI scores in CD patients (Figure S8, Supporting Information). Since FUT7 mediates the synthesis of CD15s, which plays a vital role in leukocyte homing to peripheral tissues,^[^ ^15^, ^16^ ^]^ we speculated that abnormal intestinal inflammatory responses in CD may be contributed to decreased FUT7‐mediated CD15s generation in Tregs and thereby insufficient CD15s^+^ Tregs homing to the intestine. To test this hypothesis, we next determined the levels of CD15s^+^ Tregs in the blood by flow cytometry. Although the frequency of Tregs among CD4^+^ T cells was not different among the three groups, the frequency of CD15s^+^ Tregs among Tregs was significantly decreased in active CD patients, but not in quiescent CD patients, compared with healthy controls (Figure 6B–E). In addition, we detected the levels of CD15s^+^ Tregs in the colon by immunofluorescence and found that although the number of Foxp3^+^ cells was not different among the three groups, the number of CD15s^+^ Foxp3^+^ cells was significantly lower in active CD patients, but not in quiescent CD patients, than in healthy controls (Figure 6F–H).

### 2.7 WD‐Altered Gut Microbiota DownRegulate Fut7 in Tregs Through Increasing the Metabolite DCA

Because abnormal signaling crosstalk between gut microbiota and metabolites has an important role in the pathogenesis of CD, we speculated that down‐regulation of FUT7 in Tregs in patients with CD would be attributed to WD‐altered gut microbiota and metabolites. To test this hypothesis, we first characterized the microbial and metabolite compositions of fecal samples collected from RD‐ and WD‐fed mice. We found that WD effectively altered the diversity of the gut microbiota and metabolites, as reflected by the Ace and Chao index and principal coordinate analysis (PCoA) plots (Figures S9A—C and S10A, Supporting Information). Compared with RD feeding, WD feeding profoundly increased the relative abundance of *Romboutsia*, *Ileibacterium*, *Faecalibaculum*, and *Lactobacillus* (Figure S9D, Supporting Information). Metabolite classification analysis revealed that WD feeding obviously changed fecal lipids, in which 6‐Ethylchenodeoxycholic acid and deoxycholic acid (DCA) were significantly increased in the WD‐fed mice compared with the RD‐fed mice (Figure S10B,C, Supporting Information).

We subsequently performed correlation analysis of the association of the WD‐altered gut microbes and metabolites and found that 6‐Ethylchenodeoxycholic acid and DCA were positively correlated with *Lactobacillus*, *Romboutsia*, and *Eubacterium*, while negatively with *Allobaculum* and *Bifidobacterium* (Figure S10D, Supporting Information), suggesting that increases of fecal 6‐Ethylchenodeoxycholic acid and DCA in WD‐fed mice is the result of interactions among host, WD feeding, and gut microbes. Next, to explore whether the two metabolites regulate Fut7 expression in Tregs, FACS‐sorted Tregs were treated with 6‐Ethylchenodeoxycholic acid and DCA in vitro, and their Fut7 mRNA levels were determined by real‐time PCR. We found that DCA, but not 6‐Ethylchenodeoxycholic acid, significantly decreased Fut7 mRNA level in Tregs (Figure S11, Supporting Information), suggesting that DCA can downregulate Fut7 expression in Tregs. Furthermore, the limited number of Tregs available for sorting precludes their use for protein‐level detection. In subsequent in vitro experiments using the intestinal epithelial cell line NCM460, we confirmed that DCA influences FUT7 expression via its receptor S1PR2 (Figure S12, Supporting Information).

## 3 Conclusion and Discussion

This study elucidates a mechanism by which a WD disrupts the IEC barrier, primarily through impairing Tregs homing to the gut. Tregs are essential for maintaining intestinal homeostasis by modulating immune responses and supporting epithelial integrity.^[^ ^13^ ^]^ We demonstrated that WD feeding leads to a reduction in intestinal Tregs and attenuates Tregs–IEC interactions. Furthermore, we identified that WD downregulates Fut7 expression in Tregs, which is critical for their homing capacity. Fut7 encodes an α1,3‐fucosyltransferase responsible for generating functional ligands for selectins. Consistent with prior studies showing that Fut7 confers L‐, P‐, and E‐selectin binding activity and that *Fut7* ^−/−^ mice exhibit defective lymphocyte migration to inflammatory sites and lymphoid organs,^[^ ^16^, ^17^ ^]^ our data underscore the indispensable role of Fut7 in gut‐specific Treg recruitment.

Notably, we provide evidence that WD modulates Fut7 expression in Tregs via structural reshaping of the gut microbiota and subsequent elevation of the bile acid deoxycholic acid (DCA). WD feeding significantly increased the abundance of *Romboutsia *and *Lactobacillus*, which correlated positively with DCA levels. In vitro assays confirmed that DCA downregulates Fut7 expression in Tregs. Bile acids act via specific receptors, including G‐protein‐coupled bile acid receptor 5, farnesoid X receptor, pregnane X receptor, sphingosine‐1‐phosphate receptor 2, vitamin D receptor, and muscarinic receptor 2.^[^ ^19^ ^]^ In this study, we initially confirmed that DCA inhibits FUT7 expression via S1PR2. However, it is incompletely unclear which mediates DCA signaling to downregulate Fut7 expression in Tregs, which deserves further study.

To strengthen our findings, we employed TNBS‐induced colitis and naïve CD4⁺ T cell transfer model of colitis and demonstrated that Fut7 expression in Tregs is essential for restoring the IEC barrier. Fut7‐CKO mice exhibited exacerbated colitis and barrier disruption in the TNBS model, whereas Fut7 overexpression in Tregs ameliorated these defects. In the transfer model, Fut7‐knockdown Tregs failed to mitigate colitis, highlighting the necessity of Fut7 in Treg‐mediated repair. A technical limitation was the impracticality of using Fut7‐KO Tregs in transfer experiments due to the high number of donor mice required.

To our knowledge, this is the first study to report reduced FUT7 expression in Tregs from patients with active Crohn's disease (CD), suggesting that enhancing FUT7 expression may represent a novel therapeutic strategy. Indeed, nanoparticle‐mediated delivery of Fut7 (CD4‐LDP‐Fut7) specifically to Tregs significantly alleviated colitis and barrier impairment in both WD and TNBS models. However, given physiological differences between mice and humans, the safety and efficacy of such nanotherapeutic approaches in CD patients require further evaluation.

Furthermore, regarding how FUT7 deficiency specifically disrupts Treg‐IEC signaling crosstalk. We propose that this disruption operates through two interrelated mechanisms. The primary and most direct mechanism is the breakdown of physical interaction. FUT7‐synthesized CD15s form the core structure of L‐, P‐, and E‐selectin ligands on Tregs. The binding of these ligands to selectins expressed on gut microvascular endothelial cells is the critical initial step for Tregs to roll, adhere, and ultimately extravasate into the intestinal tissue.^[^ ^16^, ^17^ ^]^ Our TCR sequencing data (Figure S2, Supporting Information), showing reduced TCR similarity between colonic and peripheral blood Tregs upon WD feeding, provide direct evidence for impaired gut homing. In the absence of FUT7, Tregs fail to efficiently reach the intestinal tissue, creating a physical disconnect that precludes any potential paracrine signaling or direct cell contact with IECs. Second, we cannot exclude the possibility that FUT7 indirectly affects signaling by modulating functional surface molecules on Tregs. Beyond selectin ligands, fucosylation catalyzed by FUT7 may modify other critical immunoregulatory molecules, such as key co‐stimulatory molecules or cytokine receptors that mediate Treg contact with target cells. For instance, studies have shown that glycosylation of surface molecules like CD44 on T cells can influence their signal transduction and cellular function.^[^ ^23^ ^]^ Therefore, FUT7 deficiency may not only “lock Tregs out” but also impair the immunoregulatory function of those few Tregs that do manage to arrive, rendering them unable to effectively transmit homeostatic and pro‐repair signals to IECs, ultimately leading to disruption of the epithelial barrier. This sophisticated dual regulation of “location and function” represents a compelling avenue for future investigation.

In summary, our work reveals a pathway whereby WD disrupts intestinal barrier function via microbiota‐dependent DCA elevation, leading to Fut7 downregulation in Tregs and impaired mucosal homing. These findings provide mechanistic insights into how WD increases susceptibility to CD and propose a potential therapeutic strategy through Treg‐specific FUT7 up‐regulation using humanized FUT7 nanoparticles, which merits further preclinical and clinical investigation.

## 4 Experimental Section

### Antibodies, Reagents, and Resources

The antibodies, reagents, and resources used in this study are provided in Table S1 (Supporting Information).

### Human Samples

Peripheral blood and terminal ileal endoscopic biopsy samples were collected from patients with CD and healthy controls attending Nanfang Hospital. Disease diagnoses and activity index were assessed by a standard combination of clinical, endoscopic, and histological criteria. Peripheral blood and intestinal mucosal tissues were collected from consenting individuals during routine endoscopy according to the protocols approved by the Ethics Committee of Nanfang Hospital of Southern Medical University. Demographic characteristics are shown in Table S2 (Supporting Information).

### Mouse Models

All animal‐related experimental protocols were approved by the Institutional Animal Care and Use Committee of Southern Medical University (K2022019). Wild‐type (WT) mice (C57BL/6 strain) aged 4 weeks were assigned to regular diet (RD) and western diet (WD) models for 17 weeks. RD (AIN93G‐110700) was purchased from Dyet, and WD was prepared according to the previous study.^[^ ^19^ ^]^ The mice in the WD group were divided evenly into 3 groups. One group of mice was not treated, and the other two groups of mice were given a tail intravenous injection of the positive nanoparticle CD4‐LDP‐Fut7 and the negative nanoparticle CD4‐LDP‐NC with 240 ng g^−1^ once every 2 days for a total of three times in week 17.

*Fut7* ^fl/fl^ *foxp3* ^cre^ (CKO), and *fut7* ^fl/fl^ mice (C57BL/6 strain) were generated by Cyagen Corporation, bred, and maintained in a pathogen‐free facility, receiving standard chow and water ad libitum. The CKO mice were co‐housed with their *fut7* ^fl/fl^ littermate controls. To investigate the role of Fut7 in Tregs in the pathogenesis of CD, 8‐ to 10‐week‐old male CKO mice and their *fut7* ^fl/fl^ littermate controls were rectally injected with TNBS enema to induce a CD‐like colitis model according to a published procedure.^[^ ^24^ ^]^

When investigating the therapeutic effect of CD4‐LPD‐Fut7, WT co‐housed mice were randomly divided into four groups: WT, TNBS, TNBS+CD4‐LPD‐NC, and TNBS+CD4‐LPD‐Fut7. The TNBS‐induced colitis model was established according to a published procedure.^[^ ^24^ ^]^ Briefly, 8‐ to 10‐week‐old male WT mice were intrarectally injected with TNBS. On days 1, 2, and 3, the last two groups received intravenous tail injections of the same drug dose, 240 ng g^−1^ each time.

A naive CD4^+^ T cell adoptive transfer model was established using *Rag2* ^−/−^ mice purchased from Cyagen Biosciences (Guangzhou, China) according to a published procedure.^[^ ^21^ ^]^ Briefly, naive CD4^+^ T cells (CD4^+^ CD45RB^hi^ CD25^lo^) were sorted from the spleen of WT C57/BL6 mice by flow cytometry using the BD FACS Aria sorting flow cytometer (BD Biosciences), washed with sterile HBSS, and transferred into *Rag2^−/−^* mice by the tail vein injection for 6 weeks to construct the transfer model. Each mouse was transferred with ≈0.5× 10^6^ naive CD4^+^ T cells. To investigate the role of Fut7 in Tregs in regulating colitis in this model, FACS‐sorted splenic Tregs from WT C57/BL6 mice were cultured with Roswell Park Memorial Institute (RPMI) 1640 medium supplemented with 10 µg mL^−1^ anti‐CD3, 1 µg mL^−1^ soluble anti‐CD28, and 10 ng mL^−1^ IL‐2 at 37 °C and 5% CO_2_ for 24 h, and then transfected with *Fut7* small interfering RNA (*Fut7‐*siRNA) or negative control (nc)‐siRNA for 48 h in vitro. Subsequently, ≈0.1× 10^6^ cells were transferred into each *Rag2* ^−/−^ mouse by the tail vein injection 2 days after naive CD4^+^ T cell injection. The oligos of *Fut7‐*siRNA and nc‐siRNA are shown in Table S3 (Supporting Information).

The mice in the above models were weighed and assessed for weight loss, colon length, and their colon tissues were embedded in paraffin and stained with H&E, Occludin, and ZO‐1. Two pathologists in a double‐blind manner assessed histology scores in the colons according to the following criteria, including mucosal injury and inflammatory cell infiltration index. Mucosal injury index: 0 represents no mucosal damage; 1, mild crypt loss; 2, moderate crypt loss; 3, severe crypt loss; 4, full‐layer erosions and ulcers. Inflammatory cell infiltration index: 0 represents no evidence of inflammation; 1, mild inflammation with scattered infiltrating mononuclear cells; 2, moderate inflammation with multiple foci; 3, severe inflammation with increased vascular density; 4, transmural inflammatory cell infiltration.

### Cell Culture and Nanoparticle Delivery

FACS‐sorted splenic CD4^+^ CD25^+^ CD127^low^ Tregs and CD4^+^ CD25^−^ CD127^high^ non‐Tregs (0.5× 10^5^ cells per well) from C57BL/6 mice were cultured with RPMI 1640 medium supplemented with 10 µg mL^−1^ anti‐CD3, 1 µg mL^−1^ soluble anti‐CD28, and 10 ng mL^−1^ IL‐2 at 37 °C and 5% CO_2_ for 24 h, and then treated with CD4‐LDP‐Fut7 and CD4‐LDP‐NC at a concentration of 5 µg mL^−1^ for 48 h in vitro. Subsequently, the fluorescence images were obtained by a fluorescence microscope.

### Quantitative Real‐Time PCR

Total RNA was isolated using AG RNAex Pro RNA extraction reagent (Accurate Biology, China), then reverse transcribed using Evo M‐MLV reverse transcription premix kit (Accurate Biology, China). Real‐time quantitative reverse transcription PCR (qRT‐PCR) was then performed with a SYBR mix (Accurate Biology, China) via the following cycling: 95 °C for 30 s, then 40 cycles of 95 °C for 5 s, 55 °C for 30 s, and 72 °C for 30 s. A Roche LightCycle@ 480II system was used for this reaction. Relative mRNA expression levels were obtained based on normalization against GAPDH. The fold changes in expression were determined via 2^−△△CT^ calculations. The primers used for the target genes are shown in Table S3 (Supporting Information).

### Tissue Preparation and Cell Isolation

The mouse spleen was separated and placed on a 70 µm stainless steel net in a 1640 medium plate. The splenic cell suspension was obtained by gently pressing the 1.5 mL EP tube cap. After treating with erythrocyte lysate, washing, and centrifugation, the purified splenic cell suspensions were obtained and taken for subsequent experiments.

Mouse colon tissues were cleaned and segmented into 0.5 cm fragments, then transferred into 10 mL dissociation solution containing 5 mm EDTA and shaken with a horizontal shaker at 200 rpm and 37 °C for 30 min. Subsequently, colonic fragments were cleaned with PBS and incubated in a 10 mL digestive fluid system containing 1 mg mL^−1^ collagenase IV (Sigma–Aldrich, USA) and 10 mg mL^−1^ DNase I (Sigma–Aldrich, USA) on a shaker at 200 rpm and 37 °C for 30 min. After terminating with FBS, the mixture was passed through a 70‐µm cell strainer and purified with a 40% gradient Percoll solution (Cytiva, USA).

The human peripheral blood from the collection vessel was poured into a 15 mL centrifuge tube and filled with RMP1640 medium to 10 mL. Then the mixture was slowly added into a 15 mL centrifuge tube containing 4 mL Ficoll at 600 g for 25 min. The white film layer was cleaned and lysed with red blood cell lysis buffer. Finally, the purified cells were obtained after washing with RMP1640 medium.

### Flow Cytometry Analysis and Cell Sorting

For analyzing CD15s^+^ Tregs from human peripheral blood, cells were incubated with CD3‐V500, CD4‐BV605, CD8‐BV650, CD25‐PE, CD127‐BV711, and CD15S‐AF647 for 20 min, then washed and centrifuged at 1500 rpm, 5 min to remove the supernatant. Subsequently, cells were resuspended in RMP1640 medium for flow cytometry by the BD flow cytometer (BD Biosciences). For analyzing Tregs from mouse colon tissues, cells were incubated with L/D‐FVS440UV, CD45‐AF700, CD3‐APC‐CY7, CD4‐FITC, and CD25‐PE‐CY7 for 20 min, washed, and centrifuged at 1500 rpm, 5 min to remove the supernatant. Then the cells were fixed and permeabilized using lysing solution and permeabilizing solution (BD Biosciences), respectively. Next, nuclear staining with Foxp3‐eFluor 450 was performed for 45 min, and the cells were washed and centrifuged at 1500 rpm, 5 min to remove the supernatant. Subsequently, cells were resuspended in RMP1640 medium for flow cytometry by the BD flow cytometer.

For fluorescence‐activated cell sorting (FACS) of Tregs from human peripheral blood, cells were incubated with CD3‐V500, CD4‐BV605, CD8‐BV650, CD25‐PE, and CD127‐BV711 for 20 min. For sorting Tregs and non‐Tregs from mouse spleens, cells were incubated with L/D‐FVS440UV, CD45‐AF700, CD3‐APC‐CY7, CD4‐FITC, CD25‐PE‐CY7, and CD127‐BV421 for 20 min. For sorting naive CD4^+^ T cells from mouse spleens, cells were incubated with L/D‐FVS440UV, CD45‐AF700, CD3‐APC‐CY7, CD4‐FITC, and CD25‐PE‐CY7 for 20 min. Next, these cells were washed and centrifuged at 1500 rpm, 5 min to remove the supernatant. Subsequently, these cells were resuspended in RMP1640 medium with 1% penicillin–streptomycin antibiotics for sterile cell sorting by the BD FACS Aria sorting flow cytometer. The antibodies used were listed in the key resources table. In this study, fluorescence minus one (FMO) control was set to use as a basis for gating Foxp3^+^ Tregs, CD15s^+^ Tregs (Figure S13, Supporting Information).

### Intestinal Permeability Assay

Mice were given 150 µL 80 mg mL^−1^ FITC‐dextran (Sigma 46944) after a 4‐h fast. Four hours later, the mice were anesthetized, their eyeballs were removed, and their bloods were collected into an anticoagulant tube. Plasma was collected for subsequent detection after centrifugation at 3000 rpm for 10 min according to a published procedure.^[^ ^25^ ^]^ Plasma FITC‐dextran was detected using a fluorescence spectrophotometer (SpectraMax M4, Molecular Devices, USA).

### H&E Staining

Hematoxylin and eosin (H&E) staining was performed according to the previously described method.^[^ ^19^ ^]^ Briefly, colonic tissues were dissected and immediately fixed in 4% paraformaldehyde solution. Following fixation, the specimens were dehydrated, embedded in paraffin, and sectioned at a thickness of 4 µm. The tissue sections were subsequently subjected to H&E staining to assess the presence of IEC damage and crypt loss, as well as infiltration of inflammatory cells.

### Immunofluorescence Assay

Mouse colon tissues were fixed in 4% paraformaldehyde for 24 h and embedded in paraffin. Then a 4 µm‐thick colon tissue sections were cut for immunofluorescence. After dewaxing paraffin sections to water, antigen repair, blocking, and sealing, colon sections were incubated with a primary antibody solution of Occludin and ZO‐1 overnight at 4 °C, then selected the second antibody corresponding to the primary antibody using a method of tyramide signal amplification. The levels of Occludin and ZO‐1 were captured by a Pannoramic SCAN (3DHISTECH CaseViewer, Hungary). The mean fluorescence intensity (MFI) was quantified by ImageJ software. The antibodies used were listed in the key resources table.

### TCR‐Sequencing Analysis

Colorectum single cells and PBMC were isolated from littermate wild‐type mice fed with a regular normal diet (RD) and a Western diet (WD) for 16 weeks, and then their TCR‐sequencing analysis was performed by 10K Genomics, Shanghai, China.

### Spatial Transcriptomic Analysis

Fresh tissues were simultaneously frozen and embedded in optical cutting tissue (OCT) compound using liquid nitrogen. The RNA quality of the OCT‐embedded blocks was evaluated using an Agilent 2100 system. To optimize permeabilization conditions for the tissue, the Visium Spatial Tissue Optimization Slide & Reagent kit from 10X Genomics was employed, following the guidelines provided in the Visium Spatial Tissue Optimization User Guide (CG000238, 10X Genomics). For the construction of sequencing libraries, the Visium Spatial Gene Expression Slide & Reagent kit from 10X Genomics was utilized, following the protocols outlined in the Visium Spatial Gene Expression User Guide (CG000239, 10X Genomics). Sequencing was carried out on a Novaseq PE150 platform, as per the manufacturer's instructions (Illumina), with an average depth of 300 million read‐pairs per sample.

An in‐house script was employed to perform basic statistical analyses on the raw data, including evaluating data quality and GC content across the sequencing cycles. The raw FASTQ files and histology images were processed on a per‐sample basis using Space Ranger software (version spaceranger‐1.2.0, 10X Genomics) with default parameters. For downstream data analysis, the filtered gene‐spot matrix and the fiducial‐aligned low‐resolution image generated by Space Ranger was utilized. The Seurat package was then applied to conduct various analyses, such as gene expression normalization, dimensionality reduction, spot clustering, and differential expression analysis. To begin, spots were filtered based on a minimum detected gene count of 100 genes. Normalization across spots was performed using the SCTransform function, and a subset of 3000 highly variable genes was selected for principal component analysis. To assess the enrichment of candidate gene sets, the clusterProfiler R package was employed, which utilized the hypergeometric distribution for statistical calculations. Pathways with a corrected p‐value below 0.05 were considered significantly enriched terms. To assign biological annotations to the enriched pathways, two pathway classification systems were utilized as reference databases for human or mouse: Gene Ontology (GO) and Kyoto Encyclopedia of Genes and Genomes (KEGG). GO annotations were retrieved from Ensembl BioMart, while KEGG pathways were obtained via the KEGG REST API. Spatial transcriptomic data analyses were performed by 10K Genomics, Shanghai, China.

### Western Blot

Western blot procedures were performed as previously described.^[^ ^26^ ^]^ The antibodies used were listed in Table S1 (Supporting Information).

### Bacterial Whole‐Length 16S rRNA Gene Amplicon Sequencing

Littermate wild‐type mice were fed with a regular normal diet and a Western diet for 16 weeks. Then their fresh fecal samples were collected and stored at −80 °C less than 30 min before DNA extraction to prevent DNA degradation and anaerobic bacteria from being exposed to oxygen. Next, bacterial genome DNA was isolated from the fecal samples by using the QIAamp DNA Mini Kit (Qiagen, Germany). Those qualified DNA samples were used to construct a library, and full‐length 16S rRNA gene amplicon sequencing and analyses were performed by Majorbio Bio‐pharm Technology Co., Ltd, Shanghai, China.

### Mass Spectrometry‐Based Metabolomics

Littermate wild‐type mice were fed with a regular normal diet and a Western diet for 16 weeks. Then their fresh fecal samples were collected and immediately stored at −80 °C. Then these samples were subjected to mass spectrometry analysis by Majorbio Bio‐pharm Technology Co., Ltd, Shanghai, China.

### Construction of CD4‐LPD‐Fut7 and CD4‐LPD‐NC

Fut7‐expressing plasmids and negative control (NC) plasmids were constructed and purchased from Kidan Biosciences Co., Ltd, Guangzhou, China. For the construction of the Fut7‐expressing plasmid, the promoter of the vector pcdna3.1 itself was cut off and replaced with the mouse Foxp3‐binding promoter sequence^[^ ^27^ ^]^ (5′‐ATAAACAA‐3′). Subsequently, the eukaryotic ribosome binding site (5′‐GCCACC‐3′), the coding sequence (CDS) of the mouse fut7 gene, and the CDS of the enhanced green fluorescent protein gene were sequentially inserted into the vector after the Foxp3‐binding promoter sequence to obtain the fut7‐expressing plasmid. The NC plasmid was constructed in the same way, and its only difference from CD4‐LDP‐Fut7 was that the Fut7 CDS was not inserted.

Cationic liposomes composed of DOTAP and Chol (1:1 molar ratio, 10 mm) were prepared by the thin film hydration method. 2 mg of DOTAP and an appropriate amount of cholesterol were dissolved in 1 mL of chloroform and evaporated at 50 °C for 2 h. Deionized water was added to hydrate the cationic lipid film after sonication in a 100 W water bath for 15 min. Then the hydrated solution passed through 400 nm, 200 nm, and 100 nm polycarbonate membranes to prepare a cationic liposome. To prepare naked LPD, 124 µL cationic liposomes, 15 µL protamine (2 mg mL^−1^), and 11 µL DEPC water were mixed as solution A and kept at room temperature (RT) for 10 min. Meanwhile, 90 µL Fut7‐expressing plasmids (or NC plasmids) (0.33 mg mL^−1^) and 60 µL DEPC water were also mixed as solution B and kept at RT for 10 min. Solutions A and B were mixed quickly to form naked LPD‐Fut7 (or LPD‐NC) and incubated at RT for 15 min. For the preparation of fluorescent liposomes, 0.1% (molar ratio) DiI was added to the lipid film.

For antibody conjugation, the antibody (CD4) was thiolated by 2‐iminothiolane at a molar ratio of 50:1. The thiolated antibody was dialyzed in distilled water for 48 h to remove excess 2‐iminothiolane. Then naked LPD‐Fut7 (or LPD‐NC) was mixed with 37.8 µL micelle solution of DSPE‐PEG‐Mal (10 mg mL^−1^) to form PEGylated LPD‐Fut7 (or LPD‐NC) at 50 °C for 10 min. 300 µg thiolated CD4 antibody was mixed with LPD‐Fut7 (or LPD‐NC) and the mixture was incubated at 4 °C overnight to form CD4‐LPD‐Fut7 or CD4‐LPD‐NC. The two nanoparticles, CD4‐LPD‐Fut7 and CD4‐LPD‐NC, were constructed and purchased from Sunlipo Biotech Research Center for Nanomedicine, Shanghai, China.

### Quantification and Statistical Analysis

All statistical analyses were conducted using GraphPad Prism 9. The Student's *t*‐test or the one‐way ANOVA was used to compare the numerical variables, and post hoc tests were performed via Tukey's test. The Kruskal‒Wallis test was used to compare nonparametric data, and post hoc tests were performed via the Steel‐Dwass test. The normality of all continuous variables was evaluated with the Shapiro‐Wilk test, using a significance level of α = 0.05 (Table S5, Supporting Information). For data that satisfied the normality assumption, the use of parametric tests (e.g., t‐test, ANOVA) were retained. For the non‐normally distributed datasets and any group comparisons involving them, non‐parametric tests (e.g., Mann‐Whitney U test, Kruskal‐Wallis test) were uniformly applied. All data are presented as the means ± standard errors of the means. *P* values < 0.05 were considered statistically significant.

## Conflict of Interest

The authors declare no conflict of interest.

## Supporting information



Supporting Information

## Data Availability

The untargeted Metabolomics data generated in this study are available at the database (https://www.ebi.ac.uk/metabolights/) under the accession number MTBLS7877. The TCR‐seq and Visium data, as well as whole‐length 16S rRNA sequencing data generated in this study, are available at the database (https://www.cncb.ac.cn/) under the accession numbers CRA011112 and CRA011071, respectively.
