# Supplementary material for: Western Diet Inhibits FUT7‐Mediated Treg Intestinal Homing to Disrupt the Homeostasis of Intestinal Epithelial Cells in Crohn's Disease
Source: Adv Sci (Weinh). 2025 Dec 15;13(13):e09541. doi: 10.1002/advs.202509541 (PMC12955945; doi:10.1002/advs.202509541)
Supplement: Supplementary file 1 — Supporting Information [file ADVS-13-e09541-s001.docx]

**Supplemental information for**

**Western diet inhibits FUT7-mediated Treg intestinal homing to disrupt the homeostasis of intestinal epithelial cells in Crohn’s disease**

This file includes Figures S1-S13 and Tables S1-S5.

**
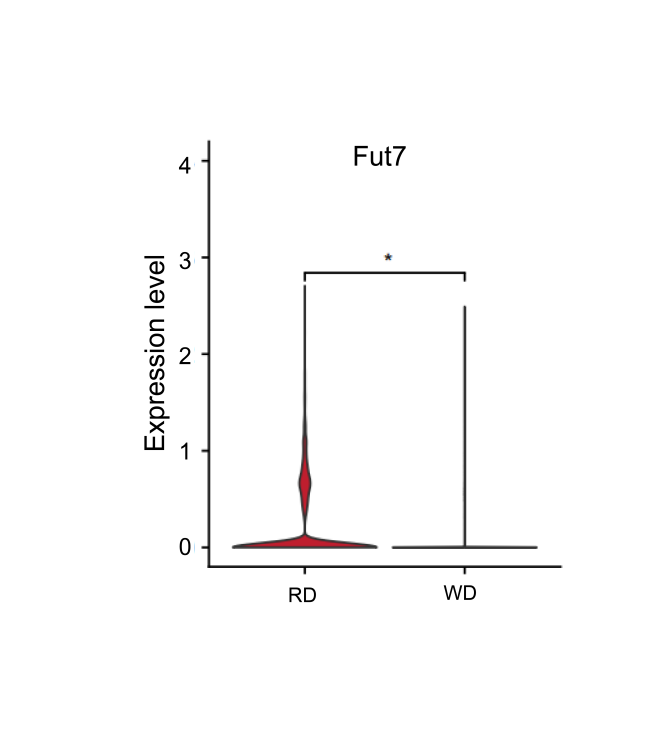
**

**Figure S1. WD upregulates Fut7 expression in Tregs.** Littermate wild-type mice were fed a regular diet (RD) or Western diet (WD) for 16 weeks and their colorectum tissues were collected for single-cell RNA sequencing analysis of Fut7 expression in Tregs. unpaired Student *t* test; **P* < 0.05.


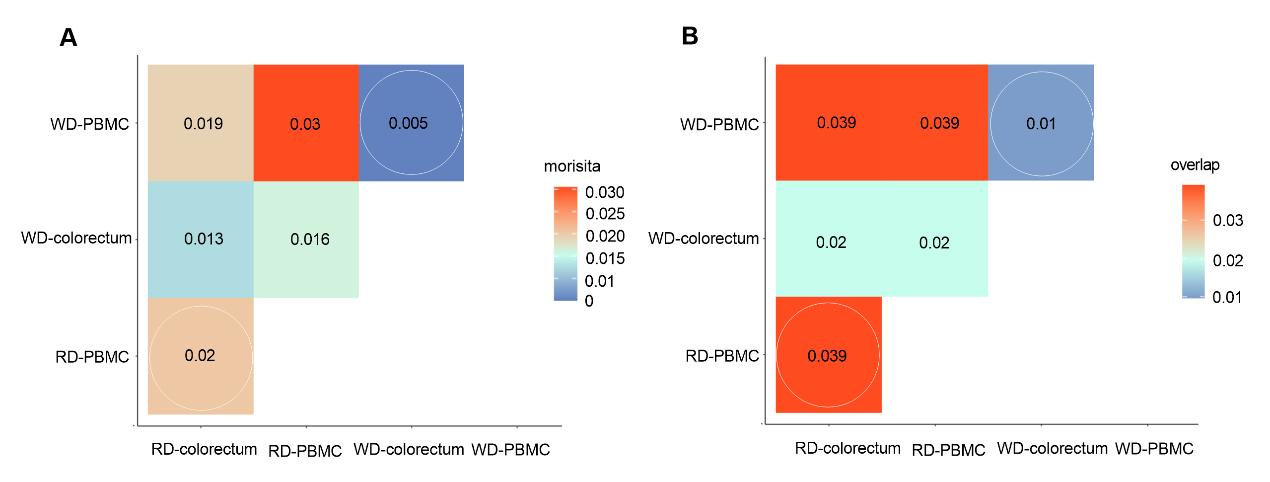


**Figure S2.** **WD decreases TCR similarity in Tregs between the gut and blood.** (*A, B*) Colorectum single cells and PBMC were isolated from littermate wild-type mice fed a regular diet (RD) or Western diet (WD) for 16 weeks, and then TCRA and TCRB in Tregs from colorectum and PBMC were analyzed and their similarity in different groups was quantified by morisita and overlap index. PBMC, peripheral blood mononuclear cell.

**
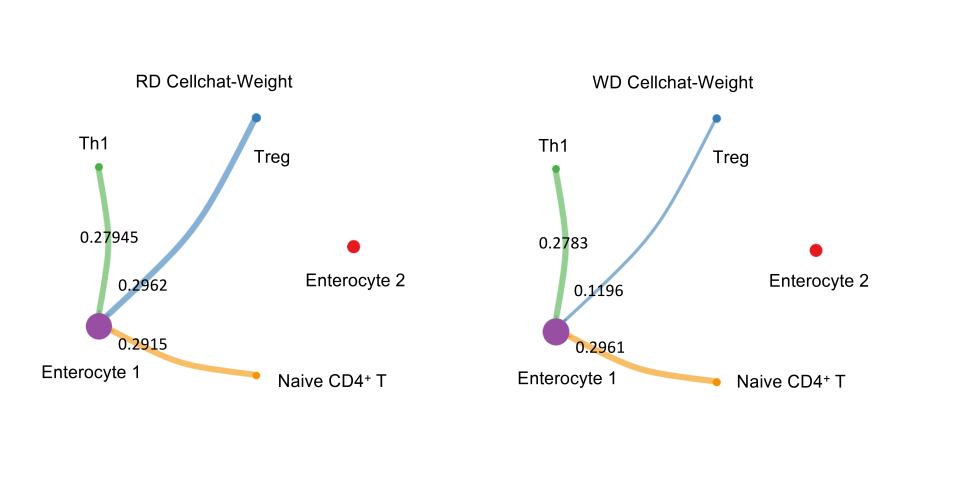
**

**Figure S3. WD downregulates the signaling dialogue from Tregs to Enterocyte 1.** Littermate wild-type mice were fed a regular diet (RD) or Western diet (WD) for 16 weeks and their colorectum tissues were collected for single-cell RNA sequencing analysis. Cell-chat analysis of signals from Tregs, and other CD4^+^ T cell subpopulations to Enterocytes.


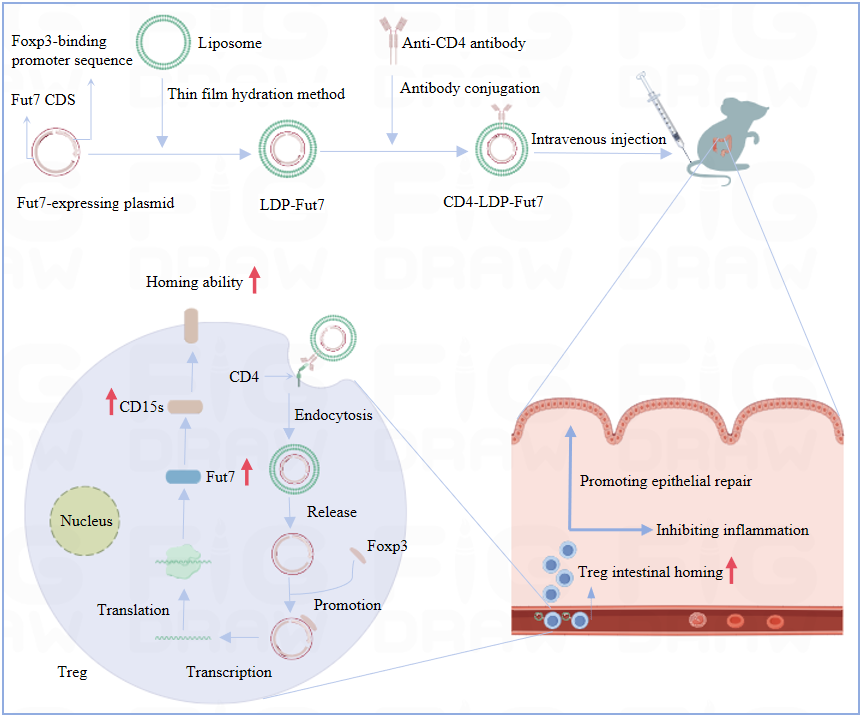


**Figure S4. Model of how CD4-LDP-Fut7 increases Treg homing to the intestine.** In this study, we constructed a nanoparticle that was CD4 antibody-mediated targeted, Fut7-expressing plasmid-loaded cationic liposome, namely CD4-LDP-Fut7. In the Fut7-expressing plasmid, the promoter of pcdna3.1 vector itself was cut off and replaced with the mouse Foxp3-binding promoter sequence, and subsequently the coding sequence (CDS) of the mouse Fut7 gene was inserted into this vector, which made CD4-LDP-Fut7 specifically target Tregs to express Fut7 gene. Upregulation of Fut7 expression in Tregs by using CD4-LDP-Fut7 can increase Treg homing to the intestine, thereby facilitating repair of the intestinal epithelial barrier and inhibiting inflammation in the intestine.


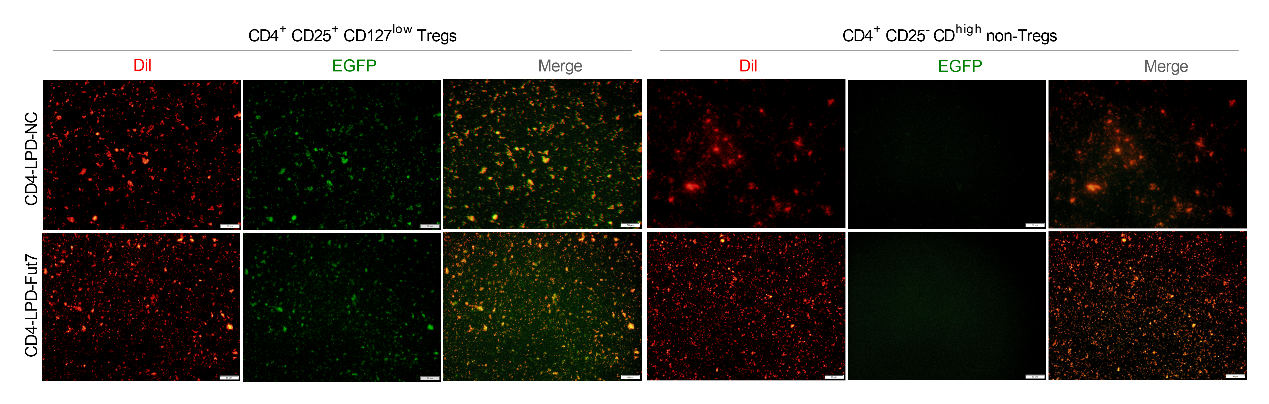


**Figure S5. Foxp3-binding promoter sequence in CD4-LPD-Fut7 and CD4-LPD-NC can be activated in Tregs.** FACS-sorted splenic CD4^+^ CD25^+^ CD127^low^ Tregs and CD4^+^ CD25^-^ CD127^high^ non-Tregs from WT mice were treated with CD4-LDP-Fut7 and CD4-LDP-NC at a concentration of 5 μg/mL for 48 h. Then, the fluorescence images were observed by a fluorescence microscope. EGFP, enhanced green fluorescent protein; FACS, fluorescence activated cell sorting; WT, wild-type.

**
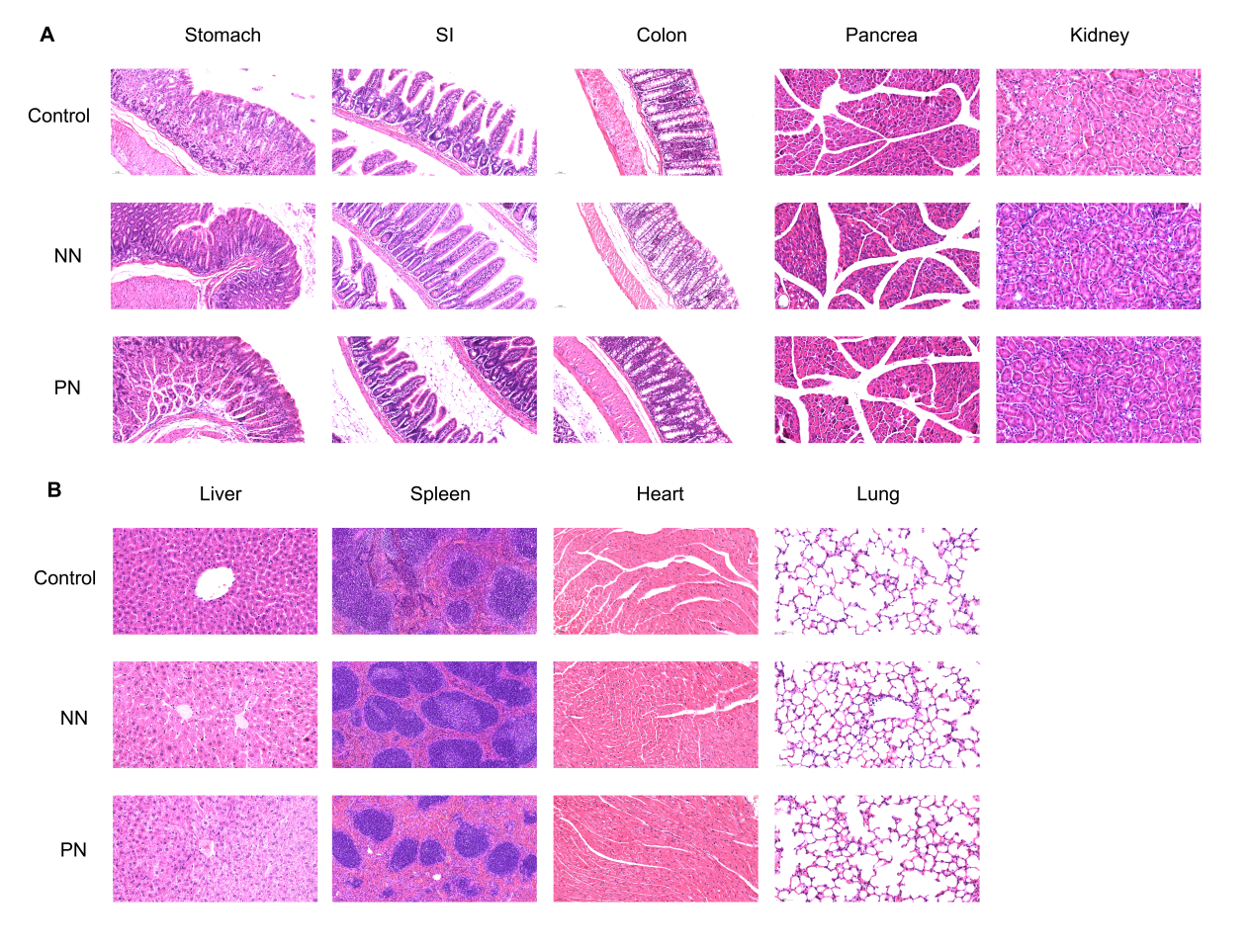
**

**Figure S6. Safety assessment of the positive nanoparticle CD4-LDP-Fut7 (PN) and the negative nanoparticle CD4-LDP-NC (NN) in *vivo*.** (*A*, *B*) Mice were given the NN or PN by intravenous tail injection. Control represents the blank control group. After 48 h later, H&E staining for multiple organs, including the stomach, small intestine (SI), colon, pancreas, kidney, liver, spleen, heart, and lung was performed to assess the safety profile of the two nanoparticles.


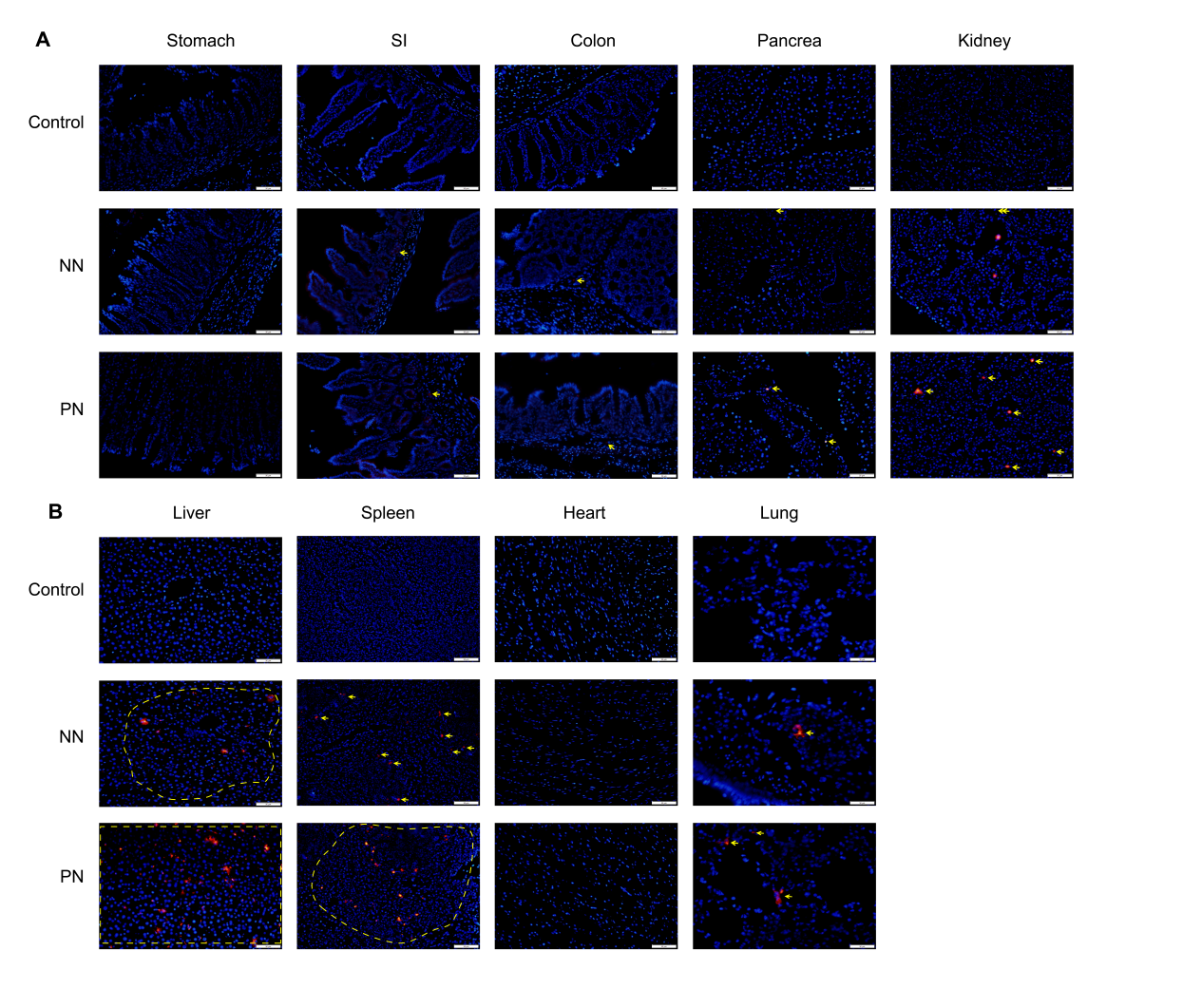


**Figure S7. Biodistribution of the positive nanoparticle CD4-LDP-Fut7 (PN) and the negative nanoparticle CD4-LDP-NC (NN) in *vivo*.** (*A*, *B*) Mice were given the NN or PN by intravenous tail injection. Control represents the blank control group. After 48 h later, carboxyfluorescein imagings of frozen organ sections from the stomach, small intestine (SI), colon, pancreas, kidney, liver, spleen, heart, and lung were examined under a fluorescence microscope.


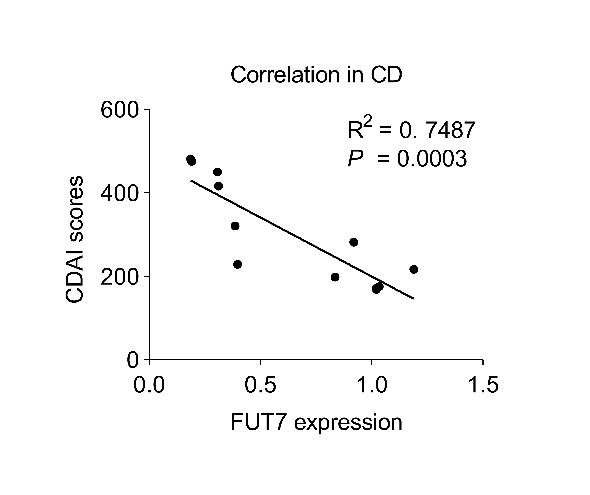


**Figure S8.** **FUT7 expression in Tregs is negatively correlated with CDAI scores in CD patients.** The mRNA levels of FUT7 in FACS-sorted Tregs from the blood of CD patients (n = 12) were detected by qPCR, and then the correlation between FUT7 levels in Tregs and CDAI scores in CD patients was calculated by Spearman correlation analysis. CDAI, Crohn’s disease activity index; FACS, fluorescence activated cell sorting.


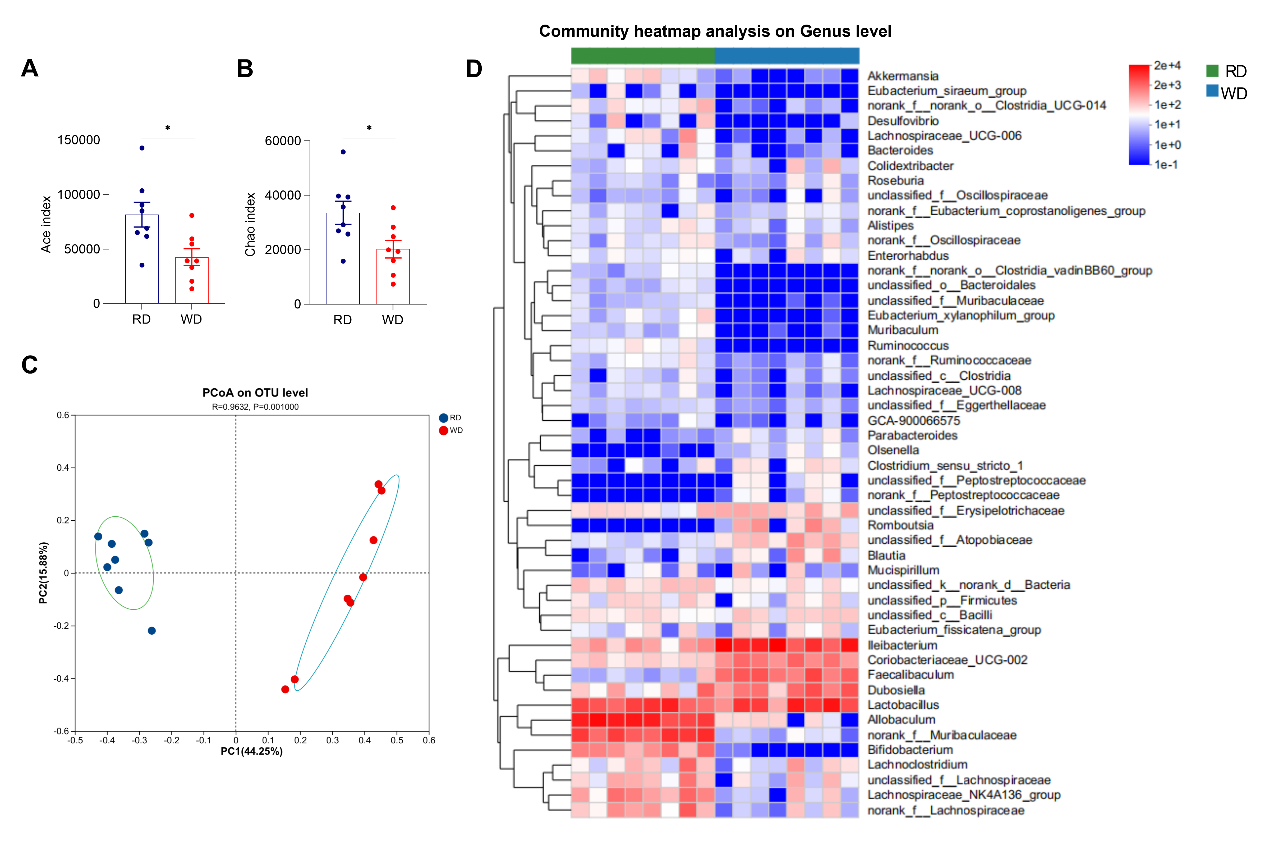


**Figure S9. WD alters microbiota compositions in the gut.** (*A to D*) Littermate wild-type mice were fed with regular normal diet (RD) and western diet (WD) for 16 weeks (n = 8/group). Then their fresh fecal were collected for genomic DNA analysis by using a 16S rRNA gene amplicon sequencing approach. (*A, B*) α-Diversity was calculated by Ace and Chao index. (*C*) β-Diversity was calculated by ordination plots based on PCoA analysis. (*D*) Community heatmap analysis on genus level.


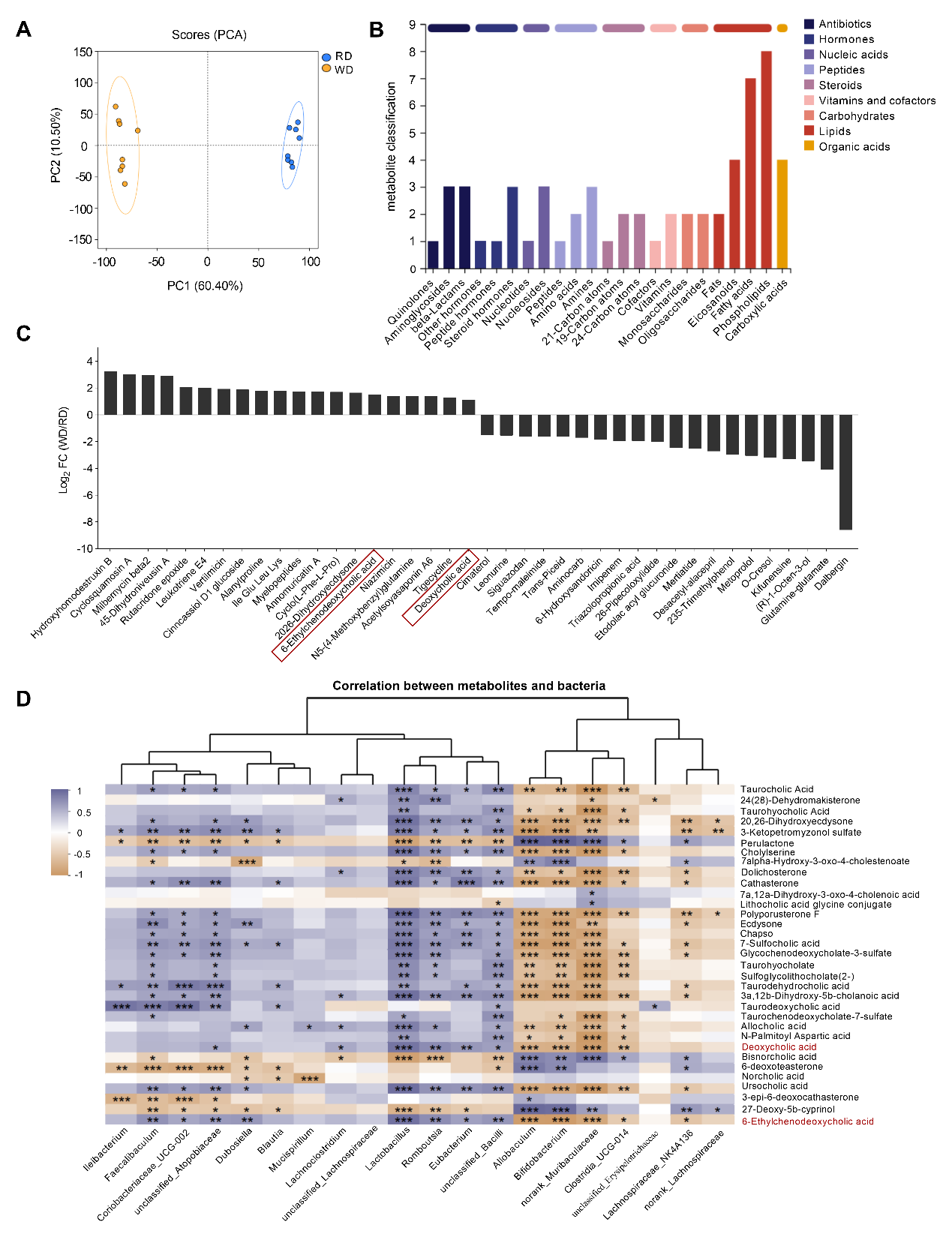


**Figure S10. WD alters metabolite compositions in the gut.** (*A to D*) Littermate wild-type mice were fed with regular normal diet (RD) and western diet (WD) for 16 weeks (n = 8/group). Then their fresh fecal were collected for metabolite composition analysis by using mass spectrometry. (*A*) Ordination plots based on PCoA analysis. (*B*) Classification of differential metabolites between the two groups. (*C*) Up- and down-regulated top 20 differential metabolites between the two groups. (*D*) Correlation analysis of the association of the WD-altered gut microbes and metabolites.


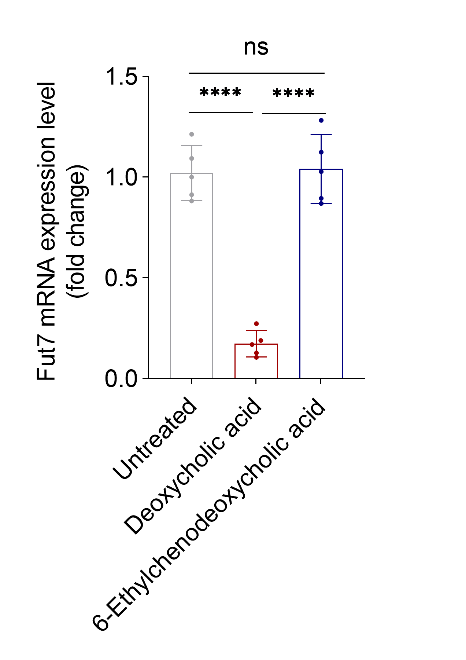


**Figure S11. DCA downregulates Fut7 expression in Tregs.** FACS-sorted splenic CD4^+^ CD25^+^ CD127^low^ Tregs from WT mice were treated with 200 μM DCA and 200 μM 6-Ethylchenodeoxycholic acid for 24 h. Then, their Fut7 mRNA levels were determined by real-time PCR. The data shown are representative of three independent experiments. One-way ANOVA; *****P* < 0.0001; ns, not significant. DCA, deoxycholic acid; FACS, fluorescence activated cell sorting; WT, wild-type.

**
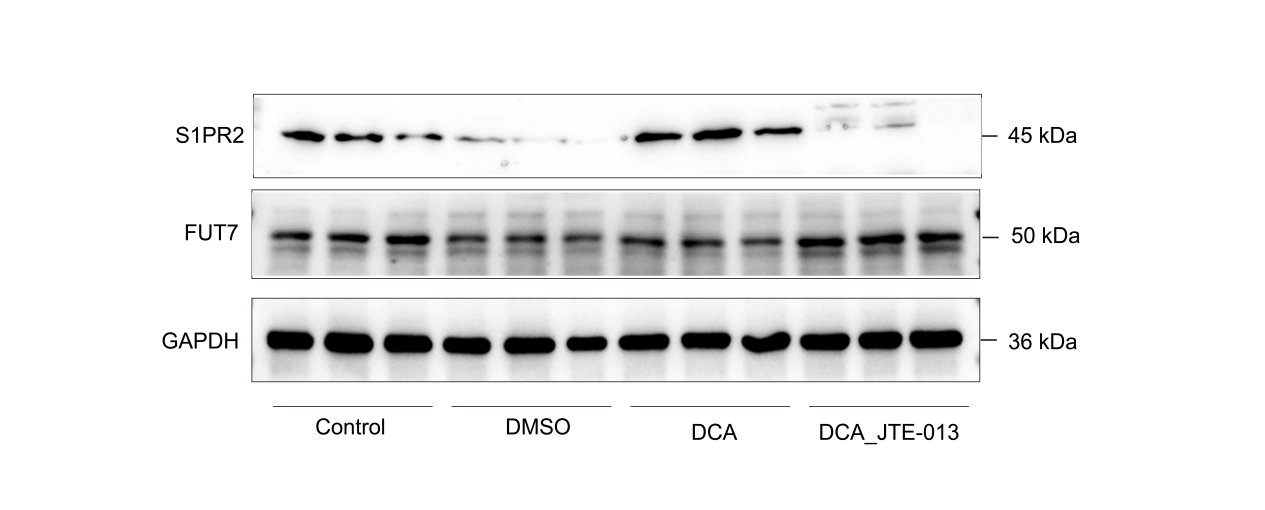
**

**Figure S12. DCA suppresses FUT7 expression through the S1PR2 pathway.** NCM460 cells were cultured in DMEM supplemented with 10% fetal bovine serum and treated with 200 μM DCA for 24 h, either in the presence or absence of the S1PR2 inhibitor JTE-013 (5 μM). Then, their indicated protein levels were determined by Western blot.


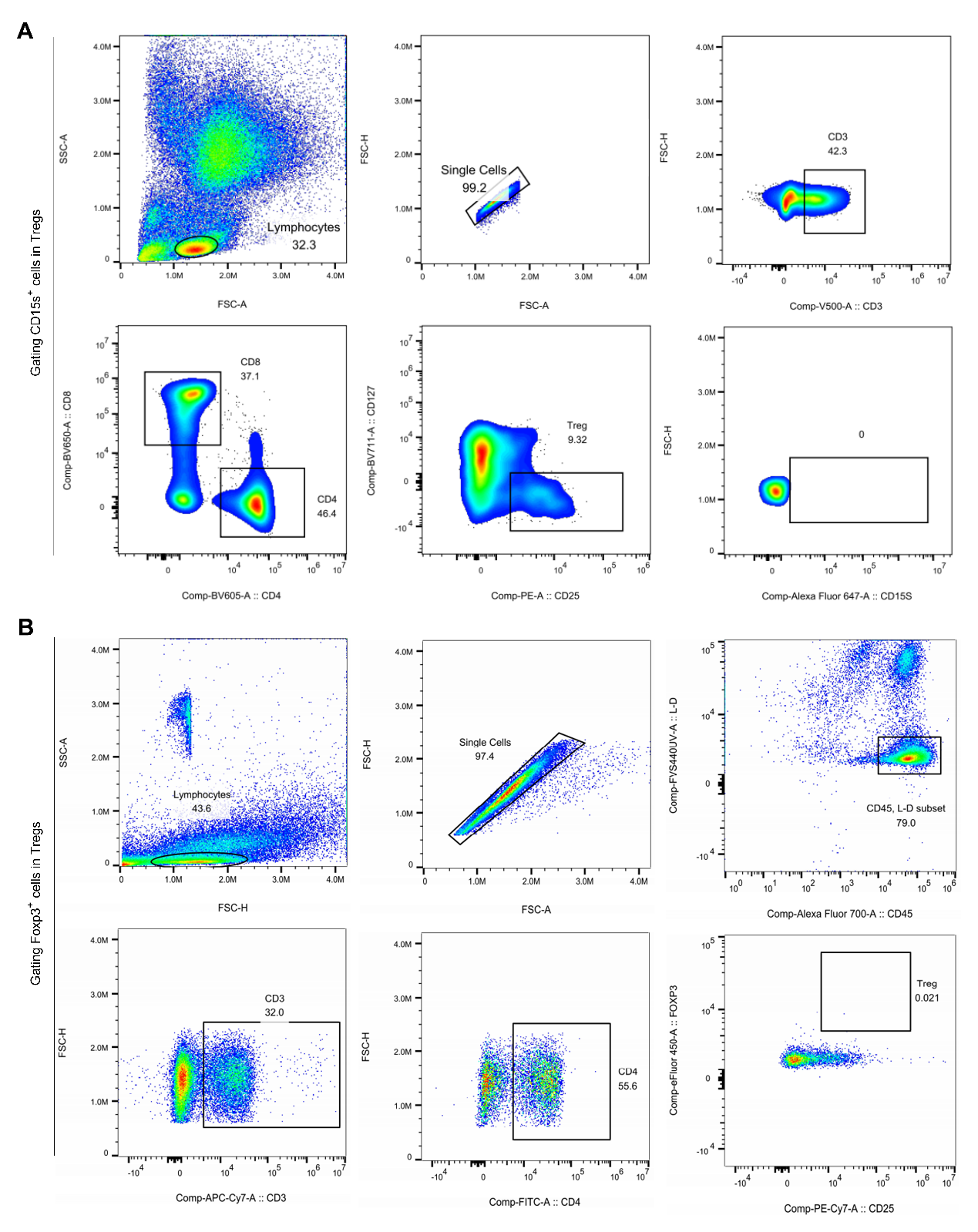


**Figure S13. Fluorescence minus one (FMO) analysis**. (*A*) Peripheral blood mononuclear cells were isolated from healthy people for FMO analysis. The FMO control was not stained with anti-CD15s antibody to use as a basis for gating CD15s^+^ Tregs. (*B*) Colonic lamina propria cells were isolated from wild-type mice for FMO analysis. This FMO control was not stained with anti-Foxp3 antibody to use as a basis for gating Foxp3^+^ Tregs.

**Table S1 Key resources**

| **REAGENT or RESOURCE** | **SOURCE** | **IDENTIFIER** |
| --- | --- | --- |
| **Antibodies** |  |  |
| CD3-V500 | BD Biosciences | Cat. # 561416 |
| CD4-BV605 | BD Biosciences | Cat. # 562658 |
| CD8-BV650 | BD Biosciences | Cat. # 563822 |
| CD25-PE | BD Biosciences | Cat. # 555432 |
| CD127-BV711 | BD Biosciences | Cat. # 563165 |
| CD15S-AF647 | BD Biosciences | Cat. # 563526 |
| CD45-AF700 | BD Biosciences | Cat. # 557957 |
| L/D-FVS440UV | BD Biosciences | Cat. # 566332 |
| CD3-APC-CY7 | Biolegend | Cat. # 557596 |
| CD4-FITC | Biolegend | Cat. # 100509 |
| CD25-PE-CY7 | Biolegend | Cat. # 102016 |
| Foxp3-eFluor 450 | Thermo Fisher Scientific | Cat. # 48-5773-82 |
| CD127-BV421 | BD Biosciences | Cat. # 566377 |
| Anti-Fut7 antibody | Proteintech | Cat. # 18197-1-AP |
| Anti-Occludin antibody | Proteintech | Cat. # 27260-1-AP |
| Anti-ZO-1 antibody | Proteintech | Cat. # 21773-1-AP |
| **Chemicals and recombinant proteins** |  |  |
| RNase-free DTT | Ansiang | Cat. # 5-750 |
| Formalin | Ansiang | Cat. # 5-171 |
| Fetal bovine serum | AusGenX | Cat. # FBS500-S |
| Percoll | Cytiva | Cat. # 17089109 |
| PVDF membranes | EMD Millipore | Cat. # C3117 |
| HRP substrate | EMD Millipore | Cat. # 18-160 |
| RIPA buffer | FUDE | Cat. # FD008 |
| Eagle’s medium (DMEM) | GIBCO | Cat. # 30030 |
| ***Continued*** |  |  |
| **REAGENT or RESOURCE** | **SOURCE** | **IDENTIFIER** |
| 2,4,6-trinitrobenzenesulfonic acid (TNBS) | Sigma | Cat. # P2297 |
| Bovine Serum Albumin | Servicebio | Cat. # GC305006 |
| Paraformaldehyde | Sigma-Aldrich | Cat. # P6148 |
| Phosphate buffered saline | Solarbio | Cat. # P1010 |
| Gentle Cell Dissociation Reagent | Stemcell Technologies | Cat. # 7174 |
| EasySep™ Releasable RapidSpheres™ | Stemcell Technologies | Cat. # 50201 |
| RNase-free Water | Takara | Cat. # No.9012 |
| DAPI | Thermo Fisher Scientific | Cat. # 62248 |
| Foxp3 / Transcription Factor Staining Buffer | Thermo Fisher Scientific | Cat. # 00-5523-00 |
| JTE-013 | MedChemExpress | Cat. # HY-100675 |
| **Experimental models: organisms/strains** |  |  |
| C57BL/6 mice | This paper | N/A |
| C57BL/7 mice (fut7^fl/fl^ foxp3^cre^) | This paper | N/A |
| Rag2^-/-^ mice | This paper | N/A |
| **Critical commercial assays** |  |  |
| E.Z.N.A.® soil DNA Kit | Omega Bio-tek | Cat.# M1768 |
| RT-PCR kit | Takara | Cat.# RR014 |
| PAGE Gel Quick Preparation Kit | Yeasen | Cat.# 20324ES62 |
| Mouse Tissue Lysis Component | Yeasen | Cat.# 19697ES50 |
| Hifair® Ⅲ Reverse Transcriptase | Yeasen | Cat.# 14601ES10 |
| **Deposited data** |  |  |
| TCR-seq (colorectum and blood cells) | this manuscript | CRA011112 |
| Visium data (colorectum cells) | this manuscript | CRA011112 |
| Whole-length 16S rRNA sequencing | this manuscript | CRA011071 |
| Untargeted Metabolomics | this manuscript | MTBLS7877 |
| ***Continued*** |  |  |
| **Software and algorithms** |  |  |
| FlowJo v10.2 | BD | https://www.flowjo.com |
| ImageJ | NIH | https://imagej.nih.gov/ij/ |
| Prism software v9.0 | GraphPad Software | https://www.graphpad.com/ |
| Cell Ranger (v3.1.0) | 10x Genomics | <https://github.com/10XGenomics> |
| R (v3.6.1) | R Foundation for Statistical Computing | https://www.r-project.org |
| **REAGENT or RESOURCE** | **SOURCE** | **IDENTIFIER** |
| Seurat (v3.1.3) | Stuart et al., 2019 | <https://satijalab.org/seurat> |
| SingleCellSignalR (1.0.0) | Cabello-Aguilar et al., 2020 | https://www.bioconductor.org/ |
| Stardist | Schmidt et al., 2018 | https://github.com/stardist/stardist |

**Table S2 Demographic characteristics of the study population**

|  | **CD** | **Controls** |
| --- | --- | --- |
| Number | 46 | 23 |
| Male, n (%) | 28 (60.8%) | 12 (52.1%) |
| Age (years) | 36.72 ± 12.11 (13-62) | 36.52 ± 14.62 (15-60) |
| Disease duration (months) | 25.83 ± 20.97 (2-82) | _ |
| Age at diagnosis (year) | 23.13 ± 8.88 (13-56) | _ |

**Table S3 Primers and oligos used in this study**

|  | **Type** | **Sequences (5'-3')** |
| --- | --- | --- |
| Human FUT7 | Forward primer | CCACGATCACCATCCTTG |
|  | Reverse primer | AGGCTTCGGTTGGCACTC |
| Human GAPDH | Forward primer | ACACCCACTCCTCCACCTTTG |
|  | Reverse primer | TCCACCACCCTGTTGCTGTAG |
| Mouse Fut7 | Forward primer | CAGATGCACCCTCTAGTACTCTGG |
|  | Reverse primer | TGCACTGTCCTTCCACAACC |
| Mouse GAPDH | Forward primer | GGAGAAACCTGCCAAGTATGA |
|  | Reverse primer | TCCTCAGTGTAGCCCAAGA |
| Fut7-siRNA | Sense strand | GCAUGAAUGAGAGUCGUUATT |
|  | Antisense strand | UAACGACUCUCAUUCAUGCTT |
| NC-siRNA | Sense strand | UUCUCCGAACGUGUCACGUTT |
|  | Antisense strand | ACGUGACACGUUCGGAGAATT |

**Table S4 Characterization of nanocarriers**

| Nanoparticles | Size (nm) | Zeta potential (mv) | Plasmid EE*(%) |
| --- | --- | --- | --- |
| CD4-LPD-Fut7  CD4-LPD-NC | 189.3 ± 11.4  179.4 ± 9.6 | +5.6 ± 0.5  +5.9 ± 0.9 | 96.2 ± 3.8  96.8 ± 2.9 |

*EE: encapsulation efficacy. Data are expressed as mean ± SD (n = 3).

**Table S5 Results of Normality Tests for Continuous Variables**

| **Variable** | **Group** | **Sample Size (n)** | **Shapiro-Wilk Statistic (W)** | **p-value** | **Normal** |
| --- | --- | --- | --- | --- | --- |
| Figure 1. FITC | RD | 8 | 0.9151 | 0.3914 | Yes |
|  | WD | 8 | 0.9641 | 0.8477 | Yes |
|  | WD+PN | 8 | 0.9107 | 0.3587 | Yes |
|  | WD+NN | 8 | 0.8983 | 0.2791 | Yes |
| Figure 1. Fut7 mRNA | RD | 5 | 0.933 | 0.617 | Yes |
|  | WD | 5 | 0.8568 | 0.2168 | Yes |
|  | WD+PN | 5 | 0.9634 | 0.8313 | Yes |
|  | WD+NN | 5 | 0.8024 | 0.0847 | Yes |
| Figure 1. Colon length | RD | 8 | 0.9146 | 0.3873 | Yes |
|  | WD | 8 | 0.9674 | 0.8771 | Yes |
|  | WD+PN | 8 | 0.8461 | 0.087 | Yes |
|  | WD+NN | 8 | 0.9695 | 0.8944 | Yes |
| Figure 1. Treg | RD | 6 | 0.9095 | 0.4329 | Yes |
|  | WD | 6 | 0.9402 | 0.6605 | Yes |
|  | WD+PN | 6 | 0.9718 | 0.9045 | Yes |
|  | WD+NN | 6 | 0.8044 | 0.0644 | Yes |
| Figure 1. Mucosal injury | RD | 8 | 0.5659 | <0.0001 | No |
|  | WD | 8 | 0.9173 | 0.4082 | Yes |
|  | WD+PN | 8 | 0.8489 | 0.0929 | Yes |
|  | WD+NN | 8 | 0.9056 | 0.3241 | Yes |
| Figure 1. Inflammatory cell infiltration | RD | 8 | 0.4184 | <0.0001 | No |
|  | WD | 8 | 0.9116 | 0.3657 | Yes |
|  | WD+PN | 8 | 0.8352 | 0.0672 | Yes |
|  | WD+NN | 8 | 0.9307 | 0.5224 | Yes |
| Figure 1. Occludin | RD | 6 | 0.9447 | 0.6975 | Yes |
|  | WD | 8 | 0.9587 | 0.798 | Yes |
|  | WD+PN | 8 | 0.9135 | 0.3794 | Yes |
|  | WD+NN | 8 | 0.941 | 0.6209 | Yes |
| Figure 1. ZO-1 | RD | 6 | 0.9476 | 0.7211 | Yes |
|  | WD | 8 | 0.9204 | 0.4335 | Yes |
|  | WD+PN | 8 | 0.8517 | 0.0991 | Yes |
|  | WD+NN | 8 | 0.9593 | 0.8038 | Yes |
| Figure 3. Fut7 mRNA | Untreated | 6 | 0.9366 | 0.6322 | Yes |
|  | nc-siRNA | 6 | 0.9666 | 0.8689 | Yes |
|  | Fut7-siRNA | 6 | 0.9531 | 0.7653 | Yes |
| Figure 3. Weight loss | 1 | 6 | 0.9095 | 0.4332 | Yes |
|  | 2 | 8 | 0.8721 | 0.1581 | Yes |
|  | 3 | 8 | 0.9429 | 0.6396 | Yes |
|  | 4 | 8 | 0.9015 | 0.2983 | Yes |
| Figure 3. Colon length | 1 | 6 | 0.9763 | 0.9316 | Yes |
|  | 2 | 8 | 0.85 | 0.0953 | Yes |
|  | 3 | 8 | 0.9534 | 0.7459 | Yes |
|  | 4 | 8 | 0.9226 | 0.4513 | Yes |
| Figure 3. FITC | 1 | 6 | 0.8378 | 0.1249 | Yes |
|  | 2 | 8 | 0.8039 | 0.0315 | No |
|  | 3 | 8 | 0.8805 | 0.1903 | Yes |
|  | 4 | 8 | 0.7815 | 0.0181 | No |
| Figure 3. Mucosal injury | 1 | 6 | 0.4961 | <0.0001 | No |
|  | 2 | 8 | 0.8489 | 0.0929 | Yes |
|  | 3 | 8 | 0.8352 | 0.0672 | Yes |
|  | 4 | 8 | 0.8104 | 0.037 | No |
| Figure 3. Inflammatory cell infiltration | 1 | 6 | 0.6399 | 0.0014 | No |
|  | 2 | 8 | 0.8352 | 0.0672 | Yes |
|  | 3 | 8 | 0.8272 | 0.0555 | Yes |
|  | 4 | 8 | 0.9056 | 0.3241 | Yes |
| Figure 3. Occludin | 1 | 6 | 0.9413 | 0.6695 | Yes |
|  | 2 | 8 | 0.9343 | 0.5557 | Yes |
|  | 3 | 8 | 0.9116 | 0.3653 | Yes |
|  | 4 | 8 | 0.9241 | 0.464 | Yes |
| Figure 3. ZO-1 | 1 | 6 | 0.8847 | 0.2914 | Yes |
|  | 2 | 8 | 0.9509 | 0.7204 | Yes |
|  | 3 | 8 | 0.8319 | 0.0622 | Yes |
|  | 4 | 8 | 0.9165 | 0.4019 | Yes |
| Figure 4. Colon length | fl/fl PBS | 6 | 0.9879 | 0.9835 | Yes |
|  | CKO PBS | 6 | 0.9868 | 0.9801 | Yes |
|  | fl/fl TNBS | 8 | 0.9879 | 0.9912 | Yes |
|  | CKO TNBS | 8 | 0.9879 | 0.9912 | Yes |
| Figure 4. Inflammation | fl/fl PBS | 6 | _ | _ | _ |
|  | CKO PBS | 6 | _ | _ | _ |
|  | fl/fl TNBS | 8 | 0.8104 | 0.037 | No |
|  | CKO TNBS | 8 | 0.6412 | 0.0005 | No |
| Figure 4. Colonic Tregs in CD4+T cells (%) | fl/fl | 6 | 0.8667 | 0.2133 | Yes |
|  | CKO | 6 | 0.9616 | 0.8319 | Yes |
| Figure 4. Weight loss | fl/fl PBS | 6 | 0.8774 | 0.2574 | Yes |
|  | CKO PBS | 6 | 0.8566 | 0.1777 | Yes |
|  | fl/fl TNBS | 8 | 0.9299 | 0.5153 | Yes |
|  | CKO TNBS | 8 | 0.9699 | 0.8975 | Yes |
| Figure 4. Mucosal injury | fl/fl PBS | 6 | _ | _ | _ |
|  | CKO PBS | 6 | _ | _ | _ |
|  | fl/fl TNBS | 8 | 0.9116 | 0.3657 | Yes |
|  | CKO TNBS | 8 | 0.7238 | 0.0042 | No |
| Figure 4. FITC | fl/fl PBS | 6 | 0.9068 | 0.4156 | Yes |
|  | CKO PBS | 6 | 0.9483 | 0.7265 | Yes |
|  | fl/fl TNBS | 8 | 0.9652 | 0.8581 | Yes |
|  | CKO TNBS | 8 | 0.9129 | 0.3746 | Yes |
| Figure 5. Weight loss | fl/fl PBS | 8 | 0.8165 | 0.0429 | No |
|  | CKO PBS | 8 | 0.9276 | 0.4948 | Yes |
|  | fl/fl TNBS | 8 | 0.9666 | 0.8699 | Yes |
|  | CKO TNBS | 8 | 0.9121 | 0.3692 | Yes |
| Figure 5. Colon length | fl/fl PBS | 8 | 0.8546 | 0.106 | Yes |
|  | CKO PBS | 8 | 0.8637 | 0.1308 | Yes |
|  | fl/fl TNBS | 8 | 0.9769 | 0.9458 | Yes |
|  | CKO TNBS | 8 | 0.8783 | 0.1815 | Yes |
| Figure 5. FITC | fl/fl PBS | 8 | 0.9595 | 0.8058 | Yes |
|  | CKO PBS | 8 | 0.8903 | 0.2355 | Yes |
|  | fl/fl TNBS | 8 | 0.9496 | 0.7072 | Yes |
|  | CKO TNBS | 8 | 0.9035 | 0.3104 | Yes |
| Figure 5. Mucosal injury | fl/fl PBS | 8 | _ | _ | _ |
|  | CKO PBS | 8 | 0.9173 | 0.4082 | Yes |
|  | fl/fl TNBS | 8 | 0.8272 | 0.0555 | Yes |
|  | CKO TNBS | 8 | 0.9307 | 0.5224 | Yes |
| Figure 5. Inflammation | fl/fl PBS | 8 | _ | _ | _ |
|  | CKO PBS | 8 | 0.8104 | 0.037 | No |
|  | fl/fl TNBS | 8 | 0.8272 | 0.0555 | Yes |
|  | CKO TNBS | 8 | 0.8272 | 0.0555 | Yes |
| Figure 6. Treg | Ctrl | 8 | 0.9077 | 0.3382 | Yes |
|  | Active CD | 8 | 0.9083 | 0.3419 | Yes |
|  | Quiescent CD | 8 | 0.9587 | 0.7975 | Yes |
| Figure 6. CD15S+Treg | Ctrl | 8 | 0.9048 | 0.3192 | Yes |
|  | Active CD | 8 | 0.7585 | 0.0101 | No |
|  | Quiescent CD | 8 | 0.9256 | 0.4773 | Yes |
| Figure6. Foxp3+Tregs/10*4 um^2^ | Ctrl | 9 | 0.8696 | 0.1216 | Yes |
|  | Active CD | 9 | 0.7769 | 0.0111 | No |
|  | Quiescent CD | 9 | 0.8749 | 0.1387 | Yes |
| Figure 6. CD15s+ Foxp3+Tregs/10*4 um^2^ | Ctrl | 9 | 0.9305 | 0.486 | Yes |
|  | Active CD | 9 | 0.8533 | 0.081 | Yes |
|  | Quiescent CD | 9 | 0.8994 | 0.2485 | Yes |
| Figure 6. FUT7 | Ctrl | 6 | 0.9692 | 0.8867 | Yes |
|  | Active CD | 6 | 0.8691 | 0.2227 | Yes |
|  | Quiescent CD | 6 | 0.9444 | 0.6944 | Yes |

Note: The Shapiro-Wilk test was used for normality assessment. A p-value > 0.05 indicates no significant deviation from normality. (-) represented All the observations in the group were zero.
